# Local and global energy barriers for chiral domain walls in synthetic antiferromagnet–ferromagnet lateral junctions

**DOI:** 10.1038/s41565-022-01215-z

**Published:** 2022-10-06

**Authors:** Jiho Yoon, See-Hun Yang, Jae-Chun Jeon, Andrea Migliorini, Ilya Kostanovskiy, Tianping Ma, Stuart. S. P. Parkin

**Affiliations:** 1 Max Planck Institute of Microstructure Physics, Halle, Germany; 2 Institute of Physics, Martin Luther University, Halle-Wittenberg, Halle, Germany

**Keywords:** Spintronics, Magnetic devices

## Abstract

Of great promise are synthetic antiferromagnet-based racetrack devices in which chiral composite domain walls can be efficiently moved by current. However, overcoming the trade-off between energy efficiency and thermal stability remains a major challenge. Here we show that chiral domain walls in a synthetic antiferromagnet–ferromagnet lateral junction are highly stable against large magnetic fields, while the domain walls can be efficiently moved across the junction by current. Our approach takes advantage of field-induced global energy barriers in the unique energy landscape of the junction that are added to the local energy barrier. We demonstrate that thermal fluctuations are equivalent to the magnetic field effect, thereby, surprisingly, increasing the energy barrier and further stabilizing the domain wall in the junction at higher temperatures, which is in sharp contrast to ferromagnets or synthetic antiferromagnets. We find that the threshold current density can be further decreased by tilting the junction without affecting the high domain wall stability. Furthermore, we demonstrate that chiral domain walls can be robustly confined within a ferromagnet region sandwiched on both sides by synthetic antiferromagnets and yet can be readily injected into the synthetic antiferromagnet regions by current. Our findings break the aforementioned trade-off, thereby allowing for versatile domain-wall-based memory, and logic, and beyond.

## Abstract (web summary)

Introducing local, nanoscopic ferromagnetic regions in a synthetic antiferromagnet racetrack can help to mitigate the trade-off between energy efficiency and thermal stability in magnetic domain-wall-based devices.

## Main

A domain wall (DW) is a boundary between magnetic regions (or domains) that have magnetizations oriented along distinct directions. The domains can be used to form magnetic bits in memory and logic devices^1,2^. The recent discoveries^2–4^ of several distinct current-induced torques that can be used to manipulate DWs has made possible the potential realization of high-performance magnetic racetrack memory and logic devices^5–7^. Key to these devices is the stability of the DWs. In many cases, there is an intrinsic energy barrier for their motion under the influence of a current or field. The intrinsic energy barrier for DW depinning by a current using conventional adiabatic spin-transfer torque (STT) is distinct from that by a field^8^. At the same time, the energy barrier that is relevant for thermal stability is understood to be the field-induced depinning barrier rather than that induced by current, although this has not yet been demonstrated in experiments. The driving force to depin DWs thermally is randomly fluctuating magnetic fields^9–11^ (Supplementary Note 9). However, for the case of depinning by current-induced spin–orbit torques (SOTs)^6,7^, the current and field depinning barriers are nearly identical, since there is no intrinsic pinning for the SOT case^12^. Since the SOT^13–15^ is the mainstream mechanism for manipulating chiral DWs^16–18^ due to its high efficiency and tunability, it is critical to find a new means whereby the threshold current density *J*_th_ in SOT is low while the field energy barrier is high.

**Fig. 1:**
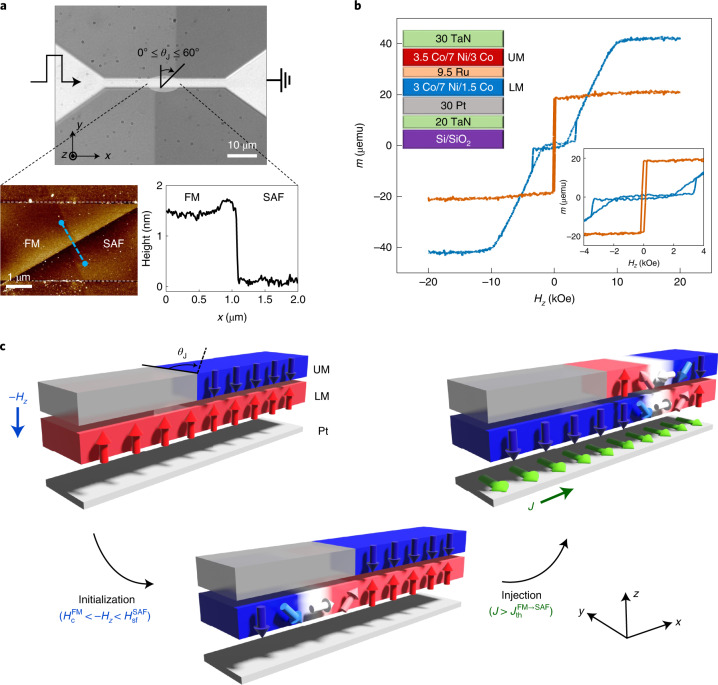
FM–SAF magnetic lateral junction. **a**, Optical (top) and atomic force microscopy (bottom) images of a representative FM–SAF junction formed within a racetrack nanowire device. The *θ*_J_ = 60°. Note that the colour contrast outside the racetrack itself originates from etching of the encapsulating alumina layer during the second resist development process (see Supplementary Information for details). The plot in the bottom right panel shows the topographic height along the blue dashed line in the bottom left panel. **b**, Magnetic easy-axis hysteresis loops, with moment *m* versus field *H*_*z*_, of the pristine SAF (blue curve) and oxidized SAF (orange curve) that is transformed into an FM. Note that the hysteresis loops are obtained from the same sample before and after the oxidation process. Bottom inset: zoomed-in view. Top inset: pristine SAF film structure consisting of the underlayer (20 TaN|/30 Pt), lower FM layer (LM; 3 Co/7 Ni/1.5 Co), exchange coupling spacer layer (9.5 Ru) and upper FM layer (UM; 3.5 Co/7 Ni/3 Co). All thicknesses are given in angstroms. **c**, Schematic illustrations of DW initialization and injection at a FM–SAF junction. By applying an external magnetic field *H*_*z*_ along the perpendicular magnetic easy-axis opposite to the initial orientation of the moment of the lower layer (LM; left), the DW is readily generated in the FM region at the junction (bottom). After initialization, the DW is injected into the SAF region by a single current pulse with a current density $J > J_{{\mathrm{th}}}^{{\mathrm{FM \to SAF}}}$ (right).

In this work, we take a new approach to resolve this long-standing critical challenge. We note that *J*_th_ and the field stability are proportional to the respective energy barrier densities to overcome the displacement of a DW from its static state. Since the corresponding energy barriers are linearly proportional to the effective DW volume, then *J*_th_ and the field stability are unchanged as the device width is shrunk to smaller dimensions. Note that both the SOT and the field-driven torques are local on the DW (Supplementary Note 13): the DW volume is typically much smaller than that of the magnetic domains themselves^19^. If we can decrease the energy barrier with respect to the effective DW volume on which the current-driven torque is applied and, at the same time, increase the energy barrier compared to the effective volume on which the field-driven torque is applied, we can overcome such a trade-off. Here we achieve this goal by forming a lateral junction between a synthetic antiferromagnet (SAF) and a ferromagnet (FM). We demonstrate that the field-driven torque is dominated by a global energy barrier that tightly binds the DW to neighbouring, large volume domains, while the current-driven torque, by contrast, is dominated by a local energy barrier defined by the DW itself. To achieve this, we employ two new concepts in the structure of the SAF–FM lateral junction. The SAF is formed from two FM layers, an upper layer and a lower layer, that are coupled antiferromagnetically with each other via a thin Ru spacer layer. By oxidizing the upper FM layer so that it becomes non-magnetic, we create lateral SAF–FM junctions, in which the lower FM layer is common to both. By design, the moment in the upper FM layer is slightly larger than that of the lower FM layer. This structure traps the DW so that depinning the DW by external fields is nearly impossible. This structure makes it possible for us to clearly demonstrate from temperature-dependent experiments that thermal effects indeed give rise to corresponding fluctuating magnetic fields. Moreover, we show from these experiments that the global energy barrier indeed increases significantly with increasing temperature. In addition, the junction is fabricated so that the boundary between the SAF and FM regions is not transverse to the length of the racetrack but rather is tilted by a junction angle *θ*_J_ (Fig. 1). This significantly reduces *J*_th_ by reducing the volume of the magnetic domain in the upper FM layer that has to be switched. Furthermore, by extending this concept to a SAF–FM–SAF bi-junction, we demonstrate that a DW can be robustly confined within the FM region of the bi-junction so that the DW is stable against arbitrary magnetic fields whose magnitudes are smaller than the coercivities of the individual SAF domains, while at the same time, the DW can be efficiently injected from the FM region into the SAF regions by current.

## SAF–FM lateral junctions

The details of sample preparation and fabrication are described in Methods. The magnetic hysteresis loops of the unpatterned SAF films exhibit a typical spin-flop transition when a field is applied perpendicular to the film along the easy magnetic axis^20^. The moments of the upper and lower FM layers are nearly compensated for, so that the remnant magnetization in zero field is almost zero. A very large magnetic field overcomes the antiferromagnet coupling to bring the upper and lower FM moments parallel to one another: the saturation moment $m_{\mathrm{s}}^{\mathrm{{SAF}}}$ is ~40 μemu with a spin-flop transition field $H_{{\mathrm{sf}}}^{\mathrm{{SAF}}}$ of ~3 kOe. The remnant moment $m_{\mathrm{R}}^{\mathrm{{SAF}}}$ is ~1 μemu. Hence, the ratio of the upper layer moment to the lower layer moment is $\frac{{m_{\mathrm{U}}}}{{m_{\mathrm{L}}}}\approx 1.05$ so that the current-driven DW motion is dominated by a giant exchange coupling torque (ECT) derived from the SAF structure^5^. The spin-flop transition shows that the antiferromagnet exchange coupling is large compared to the anisotropy of the FM layers^20^. The resist protects the upper layer of the SAF regions against oxidation during processing, as verified from magnetic hysteresis loop measurements on blanket unpatterned films (Fig. 1b). However, the FM films that are created by the oxidation of the SAF films have a coercivity $H_{\mathrm{c}}^{\mathrm{{FM}}}$ of ~0.1 kOe and a saturation moment $m_{\mathrm{s}}^{\mathrm{{FM}}}$ of ~20 µemu that is nearly half of $m_{\mathrm{s}}^{\mathrm{{SAF}}}$, that is, corresponding to the moment of the lower FM layer in the SAF. This clearly shows that the oxidation process does not affect the lower FM layer at all, while completely suppressing the magnetism of the upper layer (Fig. 1b).

## DW injection in lateral junction

**Fig. 2:**
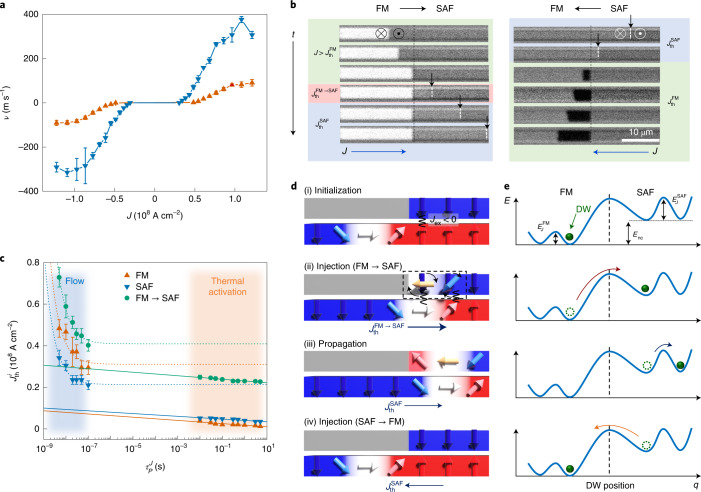
Current-driven DW motion in a FM–SAF lateral junction. **a**, DW velocity *v* versus applied current density *J* in the FM region (orange triangles) and SAF region (blue triangles). DW motion is monitored using current pulses with a temporal length $\tau _P^J = 5\,{{{\mathrm{ns}}}}$ within the FM and SAF regions. Error bars represent the standard deviation. **b**, Kerr microscope images of current-induced DW motion across the FM–SAF junction. Threshold current densities for DW motion (or injection) in each region—FM (green shaded), SAF (blue shaded), FM → SAF (red shaded) and SAF → FM (blue shaded)—are shown. The black dashed line shows the position of the junction in each panel. Here *θ*_J_ = 0°. A single DW is moved along the current flow direction by five consecutive current pulses with *J* ≈ 1.5 × 10^8^ A cm^−2^. DW positions in the SAF region are indicated by white vertical lines and black arrows. The bright and dark contrast corresponds to the domain configuration, down (↓) and up (↑), respectively. *t*, time. Light blue, red and green shaded regions correspond to the cases where the DW is located at the SAF, FM–SAF junction and FM region, respectively. The circled cross and dot symbols represent down and up domain configurations, respectively. **c**, $J_{\mathrm{{th}}}^i(i = {\mathrm{FM}},{\mathrm{FM}} \to {\mathrm{SAF}} \, {\mathrm{or}}\,{\mathrm{SAF}})$ versus current pulse length $\tau _P^J$. The flow and thermally activated regimes of DW motion are denoted by blue and orange shaded backgrounds, respectively. $J_{\mathrm{{th,flow}}}^{\mathrm{{FM}}}$ (orange triangles) and $J_{\mathrm{{th,flow}}}^{\mathrm{{SAF}}}$ (blue triangles) measured in the flow regime correspond to the current at which the DW velocity reaches ~5 m s^−1^. The $J_{\mathrm{{th}}}^i$ values in the thermally activated regime correspond to DW depinning with a probability of $P_{\mathrm{{dep}}}^i = 0.5$ (*i* = FM or SAF). For DW injection (FM → SAF), $J_{\mathrm{{th}}}^{\mathrm{{FM \to SAF}}}$ (green circles) corresponds to an injection probability of $P_{\mathrm{{inj}}}^{\mathrm{{FM \to SAF}}} = 0.5$ in both the flow and thermally activated regimes. The dashed and solid curves correspond to fits to the relation $J_{{{{\mathrm{th}},{\mathrm{flow}}}}}^i - J_{{{{\mathrm{th}}_0,{\mathrm{flow}}}}}^i \propto 1/\tau _P^J$ where *i* = FM, FM → SAF or SAF, and $J_{{{{\mathrm{th}},{\mathrm{therm}}}}}^i \propto 1 - (1/E_{{J}}^i){{{\mathrm{ln}}}}(\tau _P^J/\tau _0)$ in the flow and thermally activated regime, respectively. The error bars in the flow and thermally activated regimes correspond to the standard deviation and 25/75% probabilities, respectively. **d**,**e**, Schematic illustration of the current-driven DW injection (**d**) and corresponding energy landscape (**e**) as a function of DW position along the racetrack. Initially, the DW is located in the FM region next to the junction (i) and is injected into the SAF region by a current pulse with a magnitude that is greater than $J_{\mathrm{{th}}}^{\mathrm{{FM \to SAF}}}$ (ii). Here, the energy barrier for DW depinning in the FM region is $E_{{J}}^{\mathrm{{FM}}}$ and for DW injection is $E_{{J}}^{\mathrm{{FM \to SAF}}} = E_{\mathrm{{nc}}} + E_{{J}}^{\mathrm{{SAF}}}$, where *E*_nc_ is the nucleation energy for an additional DW in the upper FM layer of the SAF (black dashed box) and $E_{{J}}^{\mathrm{{SAF}}}$ is the DW depinning energy barrier (iii). By contrast, DW injection from the SAF into the FM region does not require any additional energy (iv).

A single chiral DW can be readily and reliably created in the FM region in a FM–SAF junction by simply applying an external field that has a value intermediate between $H_{{\mathrm{sf}}}^{\mathrm{{SAF}}}$ and $H_{\mathrm{c}}^{\mathrm{{FM}}}$, as shown schematically in Fig. 1c (Methods). The DWs move much faster in the SAF regions than in the FM regions due to the ECT^5,21–23^, which is active only in the SAF regions (Fig. 2a). Similarly, the $J_{\mathrm{{th,flow}}}^{\mathrm{{SAF}}}$ of ~3.0 × 10^7^ A cm^−2^ in the SAF regions is lower than that in the FM regions ($J_{\mathrm{{th,flow}}}^{\mathrm{{FM}}}$ of ~5.0 × 10^7^ A cm^−2^) owing to the higher torque efficiency in the SAF regions. Note that the contrast of the Kerr microscopy images in the SAF regions is significantly weaker than that in the FM regions due to the nearly compensated net moment (Fig. 2b). We find that although the DWs can be injected across the junction in both directions (FM → SAF and SAF → FM) using current pulses, the process is highly asymmetric such that $J_{{\mathrm{th,flow}}}^{\mathrm{{FM \to SAF}}} > J_{\mathrm{{th,flow}}}^{\mathrm{{FM}}} > J_{\mathrm{{th,flow}}}^{\mathrm{{SAF \to FM}}}\approx J_{\mathrm{{th,flow}}}^{\mathrm{{SAF}}}$ at *θ*_J_ = 0 (Fig. 2b). Here $J_{{\mathrm{th}},{\mathrm{flow}}}^{{\mathrm{FM}}{\rightarrow}{\mathrm{SAF}}}$, $J_{{\mathrm{th}},{\mathrm{flow}}}^{{\mathrm{FM}}}$, $J_{{\mathrm{th}},{\mathrm{flow}}}^{{\mathrm{SAF}}{\rightarrow}{\mathrm{FM}}}$ and $J_{{\mathrm{th}},{\mathrm{flow}}}^{{\mathrm{SAF}}}$ are the threshold current densities for the FM and SAF regions and FM–SAF junction in the flow regime, respectively.

The asymmetry in DW injection can be qualitatively understood as follows. First, the ECT in the SAF region gives $J_{{{{\mathrm{th}},{\mathrm{flow}}}}}^{\mathrm{{FM}}} > J_{{{{\mathrm{th}},{\mathrm{flow}}}}}^{\mathrm{{SAF}}}$ measured in the DW flow regime for which the DW depinning is dominated by the current-driven torque with no assistance from thermal fluctuations (Fig. 2c). This very efficient torque in the SAF region explains why $J_{{{{\mathrm{th}},{\mathrm{flow}}}}}^{{{{\mathrm{SAF}} \to {\mathrm{FM}}}}}\approx J_{{{{\mathrm{th}},{\mathrm{flow}}}}}^{\mathrm{{SAF}}}$ since the DW is initially created in the SAF region and has sufficient momentum to carry the DW into the FM region, where the ECT is no longer active, accounting for the higher $J_{{{{\mathrm{th}},{\mathrm{flow}}}}}^{\mathrm{{FM}}}$ in the FM region. The volume of the DW created in the FM region is reduced. On the other hand, in order to inject a DW from the FM region into the SAF region, a new DW must be nucleated near the edge of the upper FM layer in the SAF region, as shown in Fig. 2d. Hence, since such a process costs a nucleation energy *E*_nc_, $J_{{{{\mathrm{th}},{\mathrm{flow}}}}}^{{{{\mathrm{FM}} \to {\mathrm{SAF}}}}}$ is increased (with the largest increase for *θ*_J_ = 0). Another possible contribution to the energy barrier, $E_{{J}}^{{{{\mathrm{FM}} \to {\mathrm{SAF}}}}}$, is the stray field that emanates from the edge of the upper FM layer in the SAF at the junction.

## Comparison of DW energy barriers

**Fig. 3:**
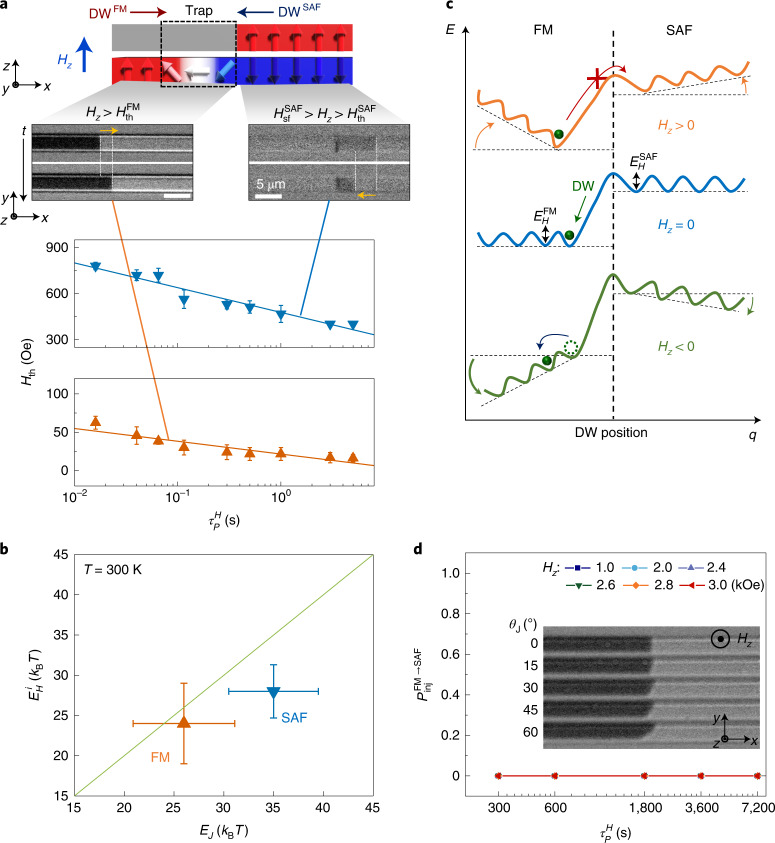
Field-driven DW motion in a FM–SAF lateral junction. **a**, Schematic illustration and magneto-optical Kerr images of field-driven DW motion within the FM region (top left) and SAF region (top right), and $\tau _P^H$-dependent $H_{\mathrm{{th}}}^i(i = {{{\mathrm{FM}}\,{{{\mathrm{or}}}}\,SAF}})$ in the DW thermally activated regime (bottom panel). The DW configuration is set to ↑↓ and its position is denoted by white vertical lines. The direction of DW motion in response to the field is depicted by yellow arrows. $H_{\mathrm{{th}}}^{\mathrm{{FM}}}$ (orange triangles) and $H_{\mathrm{{th}}}^{\mathrm{{SAF}}}$ (blue triangles) correspond to $P_{\mathrm{{inj}}}^i = 0.5$ at each $\tau _P^H$ value. Solid curves correspond to fits to $H_{\mathrm{{th}}} \propto 1 - (1/E_H){{{\mathrm{ln}}}}(\tau _P^H/\tau _0)$ to obtain $E_H^i(i = {{{\mathrm{FM}}\,{{{\mathrm{or}}}}\,SAF}})$. The error bars represent 25/75% probabilities. Scale bars, 5 µm. **b**, Plot of $E_H^i$ versus *E*_*J*_ in the thermally activated regime at *T* = 300 K. Green solid line corresponds to *E*_*J*_ = *E*_*H*_. The error bars represent the standard deviation. **c**, Schematic illustration of DW energy landscape versus DW position for various *H*_*z*_ values (*H*_*z*_ = 0, middle; *H*_*z*_ > 0, top; *H*_*z*_ < 0, bottom). The DW configuration is ↑↓. When *H*_*z*_ = 0, the DW stays in the FM region. The depinning energy barriers in the FM and SAF regions correspond to $E_H^{\mathrm{{FM}}}$ and $E_H^{\mathrm{{SAF}}}$, respectively. **d**, Plot of $P_{\mathrm{{inj}}}^{{{{\mathrm{FM}} \to {\mathrm{SAF}}}}}$ versus $\tau _H^J$ in the thermally activated regime. Inset: Kerr microscope images of DWs for 0° ≤ *θ*_J_ ≤ 60° after the application of a field up to 3 kOe for 2 h. The fact that$P_{\mathrm{{inj}}}^{{{{\mathrm{FM}} \to {\mathrm{SAF}}}}} = 0$ is shown for all *θ*_J_ and *H*_z_ values such that the different symbols all overlap.

First, we discuss the depinning of the DWs within the SAF and FM regions themselves. Detailed results for the dependence of the *J*_th_ and *H*_th_ on the corresponding pulse lengths are shown in Figs. 2c and 3a. Interestingly, when measured in the thermally activated regime, $J_{\mathrm{{th,therm}}}^{\mathrm{{SAF}}} > J_{\mathrm{{th,therm}}}^{\mathrm{{FM}}}$, while $J_{\mathrm{{th,flow}}}^{\mathrm{{SAF}}} < J_{\mathrm{{th,flow}}}^{\mathrm{{FM}}}$ when measured in the flow regime, which is technologically more useful to the development of high-speed devices (Fig. 2c). Moreover, the curve fitting in the plot of Fig. 2c shows that $E_{{J}}^{\mathrm{{SAF}}} > E_{{J}}^{\mathrm{{FM}}}$ (Fig. 3b). This is because the ECT is more effective at higher *J* values (the flow regime) than at lower *J* values (the thermally activated regime)^5^. For the field case, $H_{\mathrm{{th}}}^{\mathrm{{SAF}}} > H_{\mathrm{{th}}}^{\mathrm{{FM}}}$ and $E_H^{\mathrm{{SAF}}} > E_H^{\mathrm{{FM}}}$ in the thermally activated regime, where $H_{\mathrm{{th}}}^i$ and $E_H^i$ are the depinning fields and energy barriers for field, respectively (*i* = FM, SAF and FM → SAF; Fig. 3a). Thus, the DWs in the SAF regions are more stable against fields and thermal agitations, whereas the DWs can be more efficiently depinned by short current pulses in the SAF regions as compared to the FM regions. The physical origin of why $E_H^{\mathrm{{SAF}}} > E_H^{\mathrm{{FM}}}$ is not clear at present. It may be because (1) the DW volume of the SAF region is larger than that of the FM region or (2) the reduced net magnetization in the SAF region makes the SAF region less sensitive to the external excitations, such as fields and current pulses, in the thermally excited region, in which the ECT is not large. As summarized in Fig. 3b, a clear difference exists between the SAF and FM cases. The ratio of the energy barriers for field versus current depinning is ~1 for the FM SOT case but is considerably reduced for the SAF case.

## Confinement of DWs in lateral bi-junctions

Now we show that a DW is tightly bound near the boundary of the FM region in a FM–SAF junction when an external magnetic field *H*_*z*_ is applied (Fig. 3c; note that the SAF region is on the right-hand side of the junction, as shown in Fig. 3a). The DW sits in an energy well whose depth linearly increases with the field for *H*_*z*_ up to $H_{\mathrm{{sf}}}^{\mathrm{{SAF}}}$ (~3 kOe), as shown in Fig. 3c. This is entirely distinct from the response of DWs to fields in the interior of the FM and SAF regions (Fig. 3c). This ‘trapping’ is a consequence of the upper FM layer having a larger moment than the lower FM layer and depends on the field direction.

**Extended Data Fig. 1:**
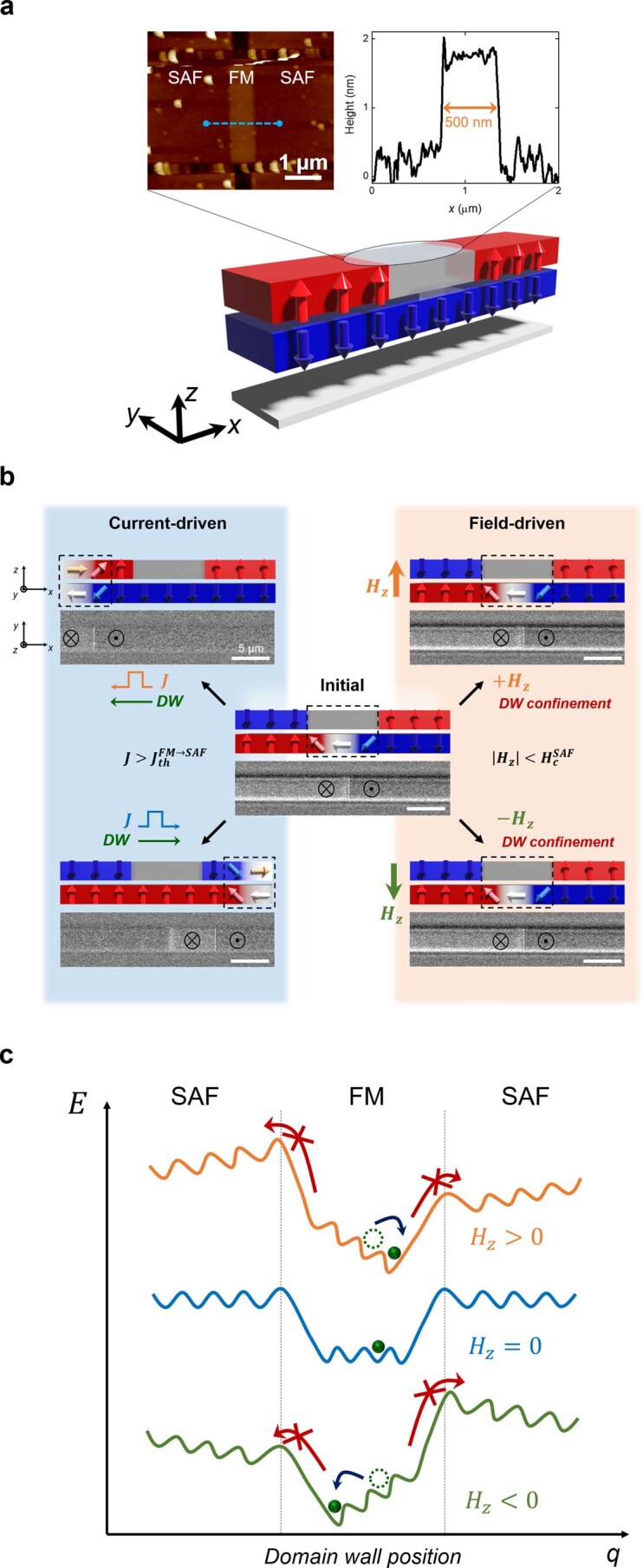
SAF-FM-SAF bi-junction. **a**, AFM image and schematic illustration of a SAF-FM-SAF lateral bi-junction. The 500 nm long FM region is sandwiched between two SAF regions. Height profile is measured by AFM across the junctions (blue dashed line). **b,c**, Schematics and Kerr microscope images of current- and field-driven DW motion within a SAF-FM-SAF bi-junction **(b)** and corresponding DW energy landscape versus position for various *H*_*z*_ (=0: middle panel, >0: top panel, <0: bottom panel) **(c)**.

For example, when a ↑↓ (up–down) configuration of DW is sitting somewhere in the FM region and the neighbouring SAF layer has the configuration of its upper layer being ↑ and its lower layer being ↓, a positive *H*_*z*_ results in the slope of energy landscape *η* being negative in the FM region but positive in the SAF region (top panel in Fig. 3c). This drives the DW towards the junction boundary, but the DW remains on the FM side of the junction. This is clearly shown in Fig. 3d where fields of up to *H*_*z*_ = 3 kOe that are applied for times of up to 2 h do not allow the DW to enter the SAF region. When the field direction is reversed, the DW moves away from the junction into the FM region, as illustrated in the bottom panel in Fig. 3c. To trap the DW for both positive and negative fields, a second junction is created with a second SAF region on the leftmost side of the FM region, thereby forming a SAF–FM–SAF bi-junction (Extended Data Fig. 1a). As shown in Extended Data Fig. 1b, experiments confirm that the DW is now firmly trapped within the FM region for |*H*_*z*_| < 3 kOe. Once a single DW is located in the FM region, the configurations of the two SAF regions are opposite to each other. Therefore, the slopes *η* of the energy landscape at the SAF–FM (left-hand side) and FM–SAF (right-hand side) junctions are tilted in opposite directions in response to external fields with opposite signs (Extended Data Fig. 1c). This gives rise to a strong confinement of the DW within the FM region. It is expected that this region can be shrunk to as little as the DW width while the trapping strength remains unchanged, thus allowing for high-density devices. This is because the domains are magnetically structureless, while the region within the DW width has only magnetic gradients, so that the device functions nearly independently of the length of the FM region until this length reaches the DW width.

## Temperature dependence of DW energy barriers

**Extended Data Fig. 2:**
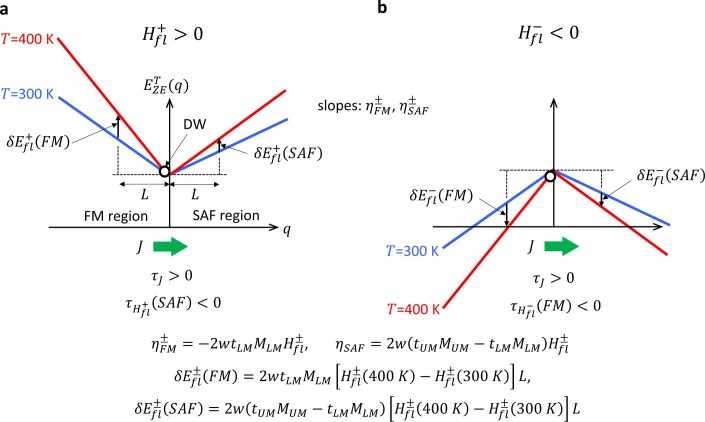
Illustration of temperature dependent energy landscapes induced by thermal fluctuation fields *H*_*fl*_ in the presence of a current pulse *J.* *J* induces a torque *τ*_*J*_ along the +*q*-direction. The DW (white circle) initially sits in the FM-region immediate next to the FM-SAF boundary. Two cases $H_{fl}^ +$ (left panel) and $H_{fl}^ -$ (right panel) that correspond to positive and negative *H*_*fl*_ along the easy *z*-axis, respectively, are considered.

First, we confirm that the current density fluctuations induced by the thermal effect in our devices are negligibly small, thereby playing no role in the DW depinning (Supplementary Note 15). Most importantly, we find from temperature-dependent experiments that thermal effects induce magnetic fluctuation fields (that is, a thermal–magnetic field equivalence) that more tightly bind DWs in the junction with increasing temperature, thus significantly increasing both the global energy barriers and the *J*_th,th__erm_ value. This is in sharp contrast with DWs in FM or SAF regions in which the thermal fluctuation fields assist in depinning DWs, thereby decreasing the *J*_th,th__erm_ value very much while keeping the energy barriers *E*_*J*_ constant. The *E*_*J*_ values for FM, SAF and the FM–SAF junction are obtained by measuring the $\tau _p^J$-dependent *J*_th__,th__erm_ value at temperature *T* = 300 K and 400 K in the absence of external magnetic fields. Note here that the *E*_*J*_ for the FM–SAF junction corresponds to the case that the DW is injected from FM region to SAF region by a current pulse along the +*q* direction, that is, the current-induced torque *τ*_*J*_ > 0 (Extended Data Fig. 2). Here *q* is the DW position. A DW with a ↑↓ configuration has been used for all the experiments, but the results are identical for the other DW configuration (↓↑).

**Fig. 4:**
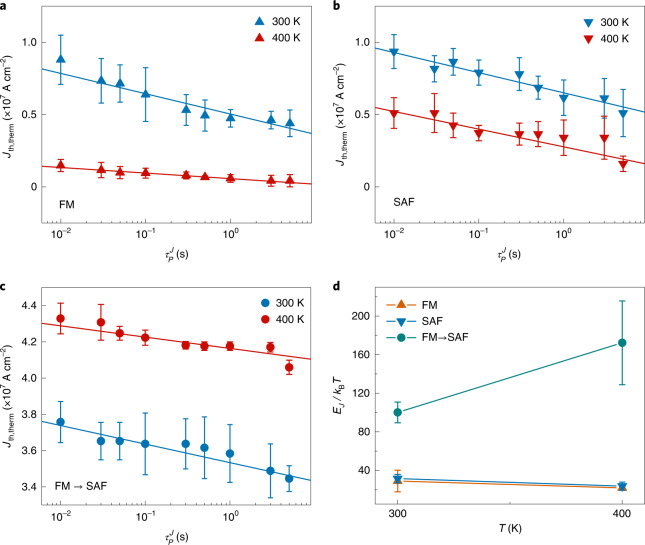
Temperature-dependent current-induced DW depinning and energy barriers. **a**–**c**, Threshold current density *J*_th__,therm_ versus current pulse length $\tau _P^J$ in the thermal activation regime at temperature *T* = 300 K (blue) and 400 K (red): FM region (**a**), SAF region (**b**) and FM–SAF junction (**c**). Solid curves correspond to the fitted ones for the $\tau_P^J$ − *J*_th,therm_ datasets. The error bars correspond to 25/75% probabilities. **d**, Energy barrier *E*_*J*_ normalized by *k*_B_*T* versus *T*. The error bars correspond to one standard deviation.

Second, we find that the $J_{\mathrm{{th,therm}}}^{\mathrm{{FM \to SAF}}}$ for the FM–SAF junction with *θ*_J_ = 0 is larger at *T* = 400 K as compared to that at *T* = 300 K (Fig. 4c and Table 1). This is in sharp contrast to what we observe for the FM (Fig. 4a) and SAF (Fig. 4b) cases, in which both $J_{\mathrm{{th,therm}}}^{\mathrm{{FM}}}$ and $J_{{\mathrm{th}},{\mathrm{therm}}}^{\mathrm{{SAF}}}$ instead decrease with increasing *T*. Note that not only the magnitude of *J*_th__,therm_ but also the slope of *J*_th__,therm_ versus $\tau _p^J$, from which the *E*_*J*_ value can be extracted, give the same result, that is, a larger *J*_th__,therm_ and a smaller slope that correspond to a larger *E*_*J*_. As clearly seen from Fig. 4c, the slope at *T* = 400 K is smaller than that at *T* = 300 K. These results thus clearly show that the *E*_*J*_ for DW injection from the FM region to the SAF region has been increased at the higher temperature, as shown in Fig. 4d. This confirms that the thermal effect induces thermal fluctuation fields *H*_fl_ that strongly trap the DW in the field-induced global energy barrier.

**Table 1:** Quantities measured from experiment and estimated from thermal fluctuations for FM, SAF and a FM–SAF junction

Quantities	FM	SAF	FM–SAF junction
*J*_th,therm_ (*T* = 300 K) (×10^6^ A cm^−2^)	8.8 ± 1.6 (10 ms)	9.4 ± 1.2 (10 ms)	38 ± 1.1 (10 ms)
*J*_th,therm_ (*T* = 400 K) (×10^6^ A cm^−2^)	1.5 ± 0.4 (10 ms)	5.1 ± 1.1 (10 ms)	43 ± 0.8 (10 ms)
*E*_*J*_/*k*_B_*T* (*T* = 300 K)	29 ± 11.2	32 ± 4.2	100 ± 10.7
*E*_*J*_/*k*_B_*T* (*T* = 400 K)	22 ± 2.3	24 ± 4.2	172 ± 43.4
*E*_*J*_ (*T* = 300 K) (eV)	0.73 ± 0.3	0.8 ± 0.11	2.5 ± 0.27
*E*_*J*_ (*T* = 400 K) (eV)	0.73 ± 0.08	0.79 ± 0.14	5.8 ± 1.44
δ*E*_*J*_ (eV)	0 ± 0.31	−0.01 ± 0.18	**3.3** ± **1.47**
$H_{\mathrm{{fl}}}^ +$ (*T* = 300 K) (Oe)	177	791	791 (SAF region)
$H_{\mathrm{{fl}}}^ -$ (*T* = 300 K) (Oe)	−177	−791	−177 (FM region)
$H_{\mathrm{{fl}}}^ +$ (*T* = 400 K) (Oe)	204	913	913 (SAF region)
$H_{\mathrm{{fl}}}^ -$ (*T* = 400 K) (Oe)	−204	−913	−204 (FM region)
$\updelta H_{\mathrm{{fl}}}^ +$ (Oe)	27	122	122 (SAF region)
$\updelta H_{\mathrm{{fl}}}^ -$ (Oe)	−27	−122	−27 (FM region)
$\updelta E_{\mathrm{{fl}}}^ +$ (eV)	4	0.9	0.9 (SAF region)
$\updelta E_{\mathrm{{fl}}}^ -$ (eV)	−4	−0.9	−4 (FM region)
$\updelta E_{\mathrm{{fl}}}^{\mathrm{{ave}}}$ (eV)	4	0.9	**2.5**

The first seven rows correspond to the quantities that are experimentally determined from the current-induced DW motion as shown in Fig. 4. The errors correspond to one standard deviation in these rows. The lower rows correspond to the quantities that are obtained from our model of thermal fluctuations (Extended Data Fig. 2). Note the bolded figures for comparison.

Next, as can be seen from Table 1, *E*_*J*_ is nearly constant as a function of *T* for the FM (*E*_*J*_ = 0.73 eV) and SAF (*E*_*J*_ = 0.79 eV) cases so that the *H*_fl_ assists in the depinning of the DWs and, correspondingly, a lower *J*_th__,therm_. These results hint that the anisotropy and magnetizations in both the FM and SAF regions are constant as *T* = 300 K → 400 K. By contrast, for the FM–SAF junction, the change of energy barrier $\updelta E_J^{\mathrm{{FM \to SAF}}} = 3.3\,{{{\mathrm{eV}}}}$.

Now let us investigate if this $\updelta E_J^{\mathrm{{FM \to SAF}}}$ quantitatively agrees with the *H*_fl_-induced global energy barrier landscape. Since *H*_fl_ points in all directions, and its easy-axis *z* component contributes only to DW depinning, let us consider two cases for the ↑↓ DW configuration: $H_{\mathrm{{fl}}}^ + > 0$ (+*z* direction) and $H_{\mathrm{{fl}}}^ - < 0$ (−*z* direction), as shown in Extended Data Fig. 2. Here the current pulse is applied along the +*q* direction such that the current-induced torque *τ*_*J*_ > 0 always drives the DW along the +*q* direction. It is assumed that the fluctuation fields are homogeneous at each instant in time, which we believe is a good approximation to describe DW depinning for the case of the junction between FM and uncompensated SAF. Importantly, the sign and magnitude of the *η* as illustrated in Extended Data Fig. 2 (red and blue lines) are determined by *H*_fl_ with the opposite signs of *η* in the FM and SAF regions. Note that the DW is sitting in the FM region immediately next to the FM–SAF boundary and not on the boundary (Extended Data Fig. 2).

Let us consider the $H_{\mathrm{{fl}}}^ +$ case. In this case, $H_{\mathrm{{fl}}}^ +$ results in $\eta _{\mathrm{{SAF}}}^ + > 0$ and $\eta _{\mathrm{{FM}}}^ + < 0$, which traps a DW in the FM region immediately next to the FM–SAF boundary (Extended Data Fig. 2a). Here $\eta _{\mathrm{{SAF}}}^ +$ and $\eta _{\mathrm{{FM}}}^ +$ correspond to the slope of energy landscape for SAF and FM regions, respectively, in the presence of $H_{\mathrm{fl}}^+$. Since $H_{\mathrm{{fl}}}^ +$ increases with increasing *T*, the magnitude of *η* increases with *T*, thus trapping the DW more tightly and increasing *J*_th__,th__erm_. Importantly, the energy landscape relevant to the DW motion is $\eta _{\mathrm{{SAF}}}^ +$, since *τ*_*J*_ (>0) tries to inject the DW into the SAF region while $\eta _{\mathrm{{SAF}}}^ +$ in the SAF region is against the injection. Consequently, the relevant energy barrier increase $\updelta E_{\mathrm{{fl}}}^ + \left( {\mathrm{{SAF}}} \right) = 0.9\,{{{\mathrm{eV}}}}$ due to $H_{\mathrm{fl}}^+$ as *T* = 300 K → 400 K when the DW trapping range *L* ≈ 100 nm is used. Here *L* ≈ 100 nm is chosen from a roughly mean value of reported pinning potential sizes: for example, ~10 nm (ref. ^9^) and ~200 nm (ref. ^24^). Note that this calculation assumes that the magnetizations do not change as *T* = 300 K → 400 K, since the *E*_*J*_ values in the FM and SAF regions are found to be constant as shown above.

Next, for the $H_{\mathrm{{fl}}}^ -$ case, $H_{\mathrm{{fl}}}^ -$ results in $\eta _{\mathrm{{SAF}}}^ - < 0$ and $\eta _{\mathrm{{FM}}}^ - > 0$ in the energy landscape, which repels a DW away from the FM–SAF junction boundary, as shown in Extended Data Fig. 2b. Since the DW is initially in the FM region, the energy landscape relevant to the DW motion is $\eta _{\mathrm{{FM}}}^ -$, which tries to move the DW along the −*q* direction, thus competing with *τ*_*J*_. This shows that $H_{\mathrm{{fl}}}^ -$ prevents the injection of a DW into the SAF region, just like in the $H_{\mathrm{{fl}}}^ +$ case. The relevant energy barrier increase is $\updelta E_{\mathrm{{fl}}}^ - \left( {\mathrm{{FM}}} \right) = 4\,{{{\mathrm{eV}}}}$ as *T* = 300 K → 400 K, when an effective DW range is *L* ≈ 100 nm.

As seen for the $H_{\mathrm{{fl}}}^ \pm$ cases above, both $H_{\mathrm{{fl}}}^ \pm$ act to form barriers against DW injection from the FM into the SAF region. Hence, the effective barrier change $\updelta E_{\mathrm{{fl}}}^{\mathrm{{ave}}}$ induced by $H_{\mathrm{{fl}}}^ \pm$ would be $\frac{{\left| {\updelta E_{\mathrm{{fl}}}^ + \left( {\mathrm{{SAF}}} \right)} \right| + \left| {\updelta E_{\mathrm{{fl}}}^ - \left( {\mathrm{{FM}}} \right)} \right|}}{2} = 2.5\,{{{\mathrm{eV}}}}$, which agrees well with the value of $\updelta E_J^{\mathrm{{FM \to SAF}}} = 3.3 \pm 1.7\,{{{\mathrm{eV}}}}$ obtained from the experiments. These results thus clearly confirm that the thermal effect is equivalent to a magnetic field in DW pinning/depinning and that the DWs are highly thermally stable in FM–SAF junctions due to the global energy barrier.

## DW injection into tilted lateral junctions

**Fig. 5:**
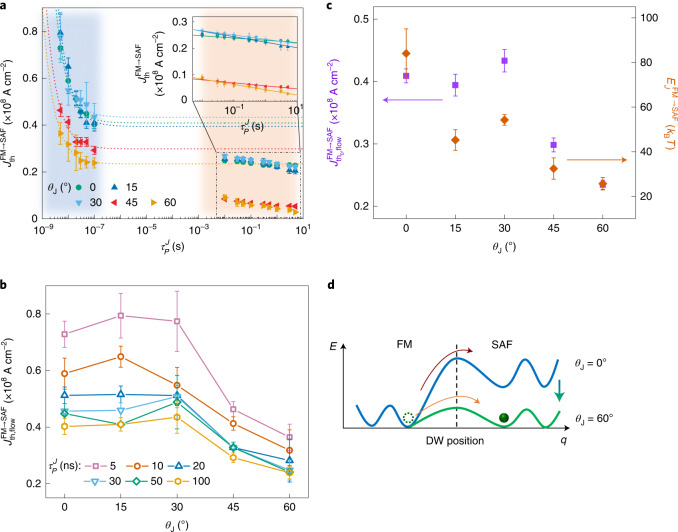
The *θ*_J_-dependent current-driven DW injection. **a**, Plot of $J_{\mathrm{{th}}}^{\mathrm{{FM \to SAF}}}$ versus $\tau _P^J$ for various *θ*_J_ values. The flow and thermally activated regimes are displayed with blue and orange shaded areas, respectively. The $J_{{{{\mathrm{th}},{\mathrm{flow}}}}}^{{{{\mathrm{FM}} \to {\mathrm{SAF}}}}}$ of *θ*_J_ = 0 (green circles), 15° (blue triangles), 30° (light blue triangles), 45° (red triangles) and 60° (yellow triangles) correspond to $P_{\mathrm{{inj}}}^{\mathrm{{FM \to SAF}}} = 0.5$. Dashed lines are fits to the flow regime with $J_{{{{\mathrm{th}},{\mathrm{flow}}}}}^{{{{\mathrm{FM}} \to {\mathrm{SAF}}}}} - J_{{{{\mathrm{th}}_0,{\mathrm{flow}}}}}^{{{{\mathrm{FM}} \to {\mathrm{SAF}}}}} \propto 1/\tau _P^J$ (ref. ^9^). Inset: zoomed-in view of the fits corresponding to the thermally activated regime $J_{{{{\mathrm{th}},{\mathrm{therm}}}}}^{{{{\mathrm{FM}} \to {\mathrm{SAF}}}}} \propto 1 - (1/E_J^{{{{\mathrm{FM}} \to {\mathrm{SAF}}}}}){{{\mathrm{ln}}}}(\tau _P^J/\tau _0)$. The error bars represent 25/75% probabilities. **b**, Plot of $\tau _P^J$-dependent $J_{{{{\mathrm{th}},{\mathrm{flow}}}}}^{{{{\mathrm{FM}} \to {\mathrm{SAF}}}}}$ versus *θ*_J_. $\tau _P^J = 5\,{{{\mathrm{ns}}}}$ (pink squares), 10 ns (orange squares), 20 ns (blue triangles), 30 ns (light blue triangles), 50 ns (green diamonds) and 100 ns (yellow hexagons) measured in the flow regime. The error bars represent 25/75% probabilities. **c**, *θ*_J_-dependent $J_{{{{\mathrm{th}}_0,{\mathrm{flow}}}}}^{{{{\mathrm{FM}} \to {\mathrm{SAF}}}}}$, purple squares; $E_J^{\mathrm{{FM \to SAF}}}$, orange diamonds. Note that $J_{{{{\mathrm{th}}_0,{\mathrm{flow}}}}}^{{{{\mathrm{FM}} \to {\mathrm{SAF}}}}}$ and $E_J^{\mathrm{{FM \to SAF}}}$ are derived from the flow regime and the thermally activated regime, respectively, using the equations in **a**. The error bars represent the standard deviation. **d**, *θ*_J_-dependent current-driven DW energy landscape. Note that $E_J^{\mathrm{{FM \to SAF}}}$ decreases with increasing *θ*_J_.

In contrast to the extreme stability of the trapped DW under a magnetic field, a DW can be expelled from the FM region into the neighbouring SAF regions by current pulses, although, as discussed above, $J_{\mathrm{{th,flow}}}^{\mathrm{{FM \to SAF}}}$ is large for *θ*_J_ = 0. Note that $J_{\mathrm{{th,flow}}}^{\mathrm{{FM \to SAF}}} > J_{\mathrm{{th,flow}}}^{\mathrm{{SAF \to FM}}}$, as discussed above. This large $J_{\mathrm{{th,flow}}}^{\mathrm{{FM \to SAF}}}$ is consistent with the large value of $E_J^{\mathrm{{FM \to SAF}}}$ of ~85*k*_B_*T* (*T* = 300 K; *k*_B_ is the Boltzmann constant) that was obtained from the thermally activated regime measurements (Fig. 5c). We find that $J_{\mathrm{{th,flow}}}^{\mathrm{{FM \to SAF}}}$ depends sensitively on *θ*_J_, decreasing with increasing *θ*_J_ for *θ*_J_ > 30 (Fig. 5b). The $J_{\mathrm{{th,flow}}}^{\mathrm{{FM \to SAF}}}$ value is nearly constant for *θ*_J_ ≤ 30, as shown in Fig. 5b. This is because the DW in the FM region aligns itself parallel to the junction boundary for small *θ*_J_ (≤30°) when a current pulse is applied. For larger *θ*_J_, this is no longer the case due to the competition between the increased DW energy versus decreased dipolar energy^25–28^. Note that the $J_{\mathrm{{th,flow}}}^{\mathrm{{FM \to SAF}}}$ value approaches that of $J_{\mathrm{{th}}}^{\mathrm{{SAF}}}$ with increasing *θ*_J_. Also we find that the dependence of $E_J^{\mathrm{{FM \to SAF}}}$ on *θ*_J_ that was obtained from thermally activated regime measurements shows a similar trend to that of $J_{\mathrm{{th,flow}}}^{\mathrm{{FM \to SAF}}}$ on *θ*_J_ (Fig. 5c). These observations are consistent with our mechanism proposed above: the effective initial nucleation volume in the upper FM layer in the SAF region decreases with increasing *θ*_J_, thereby decreasing $J_{\mathrm{{th,flow}}}^{\mathrm{{FM \to SAF}}}$ and $E_J^{\mathrm{{FM \to SAF}}}$. In addition, the propagation of the nucleated DW in the upper FM layer in the SAF region is easier the larger *θ*_J_ is, which further reduces $J_{\mathrm{{th,flow}}}^{\mathrm{{FM \to SAF}}}$ and $E_J^{\mathrm{{FM \to SAF}}}$.

## Conclusions

We have demonstrated that chiral DWs located in the FM region of a SAF–FM–SAF lateral bi-junction can be tightly bound and highly stable against large magnetic fields. However, we find that the DWs can be efficiently injected into the SAF regions across the junction by short current pulses when the *θ*_J_ is large. Here we take advantage of the localized nature of SOT and the global nature of the field-induced energy landscape: the current-induced SOT depins a DW by overcoming a local energy barrier but, on the other hand, an applied field globally tilts the energy landscape. These effects allow for a SAF–FM–SAF lateral bi-junction to induce a global energy well that tightly traps DWs. Densely packed SAF–FM–SAF lateral bi-junctions within a racetrack, which are highly thermally stable, can be used for various applications such as single-bit and multi-bit DW racetrack memory and logic^29,30^, neuromorphic devices^31,32^ and DW oscillators^33,34^.

## Methods

### Sample preparation and device fabrication

The films were grown on Si(100) wafers covered with ~250-Å-thick thermally oxidized SiO_2_ by high vacuum d.c. magnetron sputtering at an Ar pressure of 3 mtorr and in an ultra-high vacuum system with a base pressure of <10^−9^ torr. The film structure is composed of the underlayer (20 TaN/30 Pt), lower FM layer (3 Co/7 Ni/1.5 Co), exchange coupling spacer layer (9.5 Ru), upper FM layer (3.5 Co/7 Ni/3 Co) and capping layer (30 TaN), where all thicknesses are given in angstroms. Nanowires (2 µm width and 40 µm length) are formed from the SAF films using electron-beam lithography with a negative resist (AR-N 7520.18, Allergist GmbH) and Ar-ion etching. Alumina was subsequently deposited on the etched regions to protect the edges of the devices from the subsequent oxidation process that was used to define the FM regions and to form the FM–SAF junctions. For this purpose, a second electron-beam lithography step using the same negative resist was performed on top of the defined racetracks. FM regions within the SAF nanowires are formed by oxidation of the upper FM layer using an oxygen plasma treatment after patterning a resist mask on top of the nanowires. The plasma treatment is optimized so that the magnetic moment of the upper FM layer is reduced to nearly zero without affecting the moment of the lower FM layer (Supplementary Note 1). In the presence of the resist mask, plasma oxidation was carried out by reactive ion etching using an oxygen pressure of 50 mtorr and a radio-frequency power of 100 W. In this regard, we find that the Ru spacer layer acts as a passivation layer that protects the lower FM layer against oxidation even with extended oxidation times. Note that the magnetic hysteresis loop for the oxidized SAF is identical to that for a related structure formed without the upper FM layer (20 TaN/30 Pt/3 Co/7 Ni/1.5 Co/9.5 Ru/30 TaN; Supplementary Fig. 1 in Supplementary Note 1). Both the SAF and FM films show excellent perpendicular magnetic anisotropy. *θ*_J_ is varied from 0 to 60° (*θ*_J_ = 0°, 15°, 30°, 45°, 60°). The height of the FM regions in the fully patterned devices is found using atomic force microscopy to be thicker than that of the SAF regions by ~1–2 nm. This is consistent with the oxidation of the TaN capping layer, the upper FM layer and the Ru layer (Fig. 1a). The width of the FM–SAF junction, measured with atomic force microscopy, was found to be less than ~10–20 nm (Fig. 1a).

### Magnetic property measurements

Magnetic properties of blanket films were measured in a vibrating sample magnetometer (Lakeshore VSM 8600) at room temperature in the presence of an out-of-plane magnetic field varying between ±20 kOe. The influence of oxidation upon the pristine SAF unpatterned film was investigated as a function of oxidation process time, from 30 s up to 240 s. By comparison with the as-grown FM film (lower FM of SAF), the optimal oxidation time is found to be around 60 s for the SAF-to-FM transition (Supplementary Fig. 1 in Supplementary Note 1).

### X-ray photoelectron spectroscopy depth-profile analysis

Analysis of the oxidation state of the SAF and FM (oxidized SAF) films was carried out by X-ray photoelectron spectroscopy with sequential Ar-ion etching on unpatterned films. Spectra were collected using a Thermo Fisher K-alpha plus system. The X-ray photoelectron spectroscopy spectra were collected after a series of etch steps (500 eV Ar^+^ energy, 0.5 µA beam current, 5 mm × 2 mm etching area, 20 s per cycle) until the Ru peak diminishes, which indicates the completion of the etching through the upper FM layer, as shown in Supplementary Fig. 3 in Supplementary Note 3.

### Kerr microscopy and DW injection measurements

Magneto-optical Kerr microscopy was used to locate the position and tilting of the DWs within the patterned nanowire using differential mode imaging, thus allowing us to measure the DW velocity and DW injection at various temperatures ranging up to 400 K (refs. ^24–26^). These measurements were carried out in a heavily modified EVICO system. The DW injection and depinning probabilities, $P_{\mathrm{{inj}}}^{\mathrm{{FM \to SAF}}}$, $P_{\mathrm{{dep}}}^{\mathrm{{FM}}}$ and $P_{\mathrm{{dep}}}^{\mathrm{{SAF}}}$, were measured individually by monitoring the DW position for various amplitudes and lengths of current pulses and easy-axis field pulses (Supplementary Fig. 5 in Supplementary Note 5). Cases were considered in which the DW initially resides within the FM or the SAF region or at the boundary of the SAF–FM junction for various *θ*_J_ values. For the FM and SAF regions, single current pulses with lengths $\tau _P^J$ varying from 5 ns to 100 ns (flow regime) and 10 ms to 5 s (thermally activated regime) were used, while for the field pulse measurements, $\tau _P^H$ was varied from 16 ms to 5 s (thermally activated regime). Note that in the thermally activated regime, the depinning of a DW is dominated by thermal fluctuations so that the measurement in this regime is relevant to evaluate energy barriers^35^. For the reliable quantification of energy barriers, the measurements were carried out with the initial DW positioned at many different points within the FM or SAF region. The depinning measurement was then carried out ten times at each position. This procedure allowed us to avoid the effect of any local defects (Supplementary Note 7). For the measurements of DW motion across the SAF–FM junction, $\tau _P^H$ was varied from 300 s to 7,200 s. The measurements were repeated ten times in each case. The values of the energy barriers for the current and field-driven modes for the three distinct cases, $E_J^i$ and $E_H^i$ where *i* = FM, SAF or FM → SAF, were obtained from experiments in the thermally activated regime (Supplementary Note 12).

## Online content

Any methods, additional references, Nature Research reporting summaries, source data, extended data, supplementary information, acknowledgements, peer review information; details of author contributions and competing interests; and statements of data and code availability are available at 10.1038/s41565-022-01215-z.

## Supplementary information

Supplementary Information Supplementary Notes 1–15, Figs. 1–14 and refs. 1–11.

## Data Availability

The data that support the findings of this study are available from the corresponding authors upon reasonable request.
