# Supplementary material for: Local and global energy barriers for chiral domain walls in synthetic antiferromagnet–ferromagnet lateral junctions
Source: Nat Nanotechnol. 2022 Oct 6;17(11):1183–91. doi: 10.1038/s41565-022-01215-z (PMC9646530; doi:10.1038/s41565-022-01215-z)
Supplement: Supplementary file 1 — Supplementary Notes 1–15, Figs. 1–14 and refs. 1–11. [file 41565_2022_1215_MOESM1_ESM.pdf]

nature nanotechnology

Article https://doi.org/10.1038/s41565-022-01215-z

Local and global energy barriers for chiral domain walls in synthetic antiferromagnet–ferromagnet lateral junctions

In the format provided by the authors and unedited

Supplementary Information for

Local and global energy barriers for chiral domain walls in synthetic antiferromagnet-ferromagnet lateral junctions

Jiho Yoon[1,2], See-Hun Yang[1†], Jae-Chun Jeon[1], Andrea Migliorini[1], Ilya Kostanovskiy[1], Tianping Ma[1], Stuart. S. P. Parkin[1,2†]

[1]Max Planck Institute of Microstructure Physics, 06120, Halle, Germany

[2]Institute of Physics, Martin Luther University, Halle-Wittenberg, 06120, Halle, Germany

[†]Email: sehuyang@mpi-halle.mpg.de, stuart.parkin@mpi-halle.mpg.de

Supplementary Note 1. SAF to FM transition as a function of oxidation process time

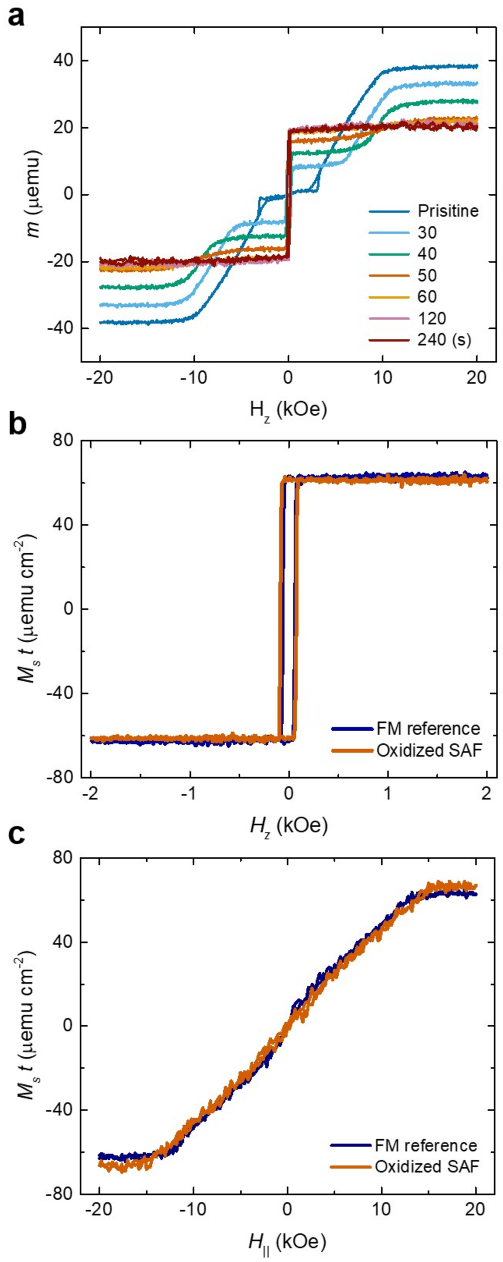

Figure S1 | Influence of oxidation upon the SAF to FM transition. a, *M-H* hysteresis loops of SAF film measured along the magnetic easy-axis as a function of oxidation process time. **b,c,** Comparison of oxidized SAF (60 sec processed) and as-grown reference FM film measured along the magnetic easy-axis (**b**) and hard-axis (**c**).

The plasma oxidation to transform SAF into FM is optimized by monitoring the magnetic hysteresis loop measured on unpatterned films using vibrating sample magnetometry. The magnetic easy axis hysteresis loops of SAF films that are treated with different oxidation times are shown in Fig. S1a. Here, the plasma oxidation is carried out using reactive ion etching (RIE) at an oxygen pressure of 50 mTorr and an RF bias power of 100 W.

The pristine SAF film exhibits a typical spin-flop transition with AF coupling and nearly zero remnant magnetization at $H_z = 0$ kOe thus showing that the SAF film is nearly compensated. The film structure is composed of the underlayer (20 TaN | 30 Pt), lower FM layer (LM : 3 Co | 7 Ni | 1.5 Co), spacer layer (9.5 Ru), upper FM layer (UM : 3.5 Co | 7 Ni | 3 Co), and capping layer (30 TaN) (all thicknesses are in Å). By increasing the oxidation time, the remnant magnetization of SAF gradually increases while the exchange field H_{ex} still remains ~ 10 kOe up to 50 sec of process time (orange line). This shows that the oxidation affects the compensated moment by reducing the magnetic moment of UM. As for 60 sec oxidation time (yellow line), the hysteresis loops shows a clear transition from SAF to FM that is evidenced by the disappearance of any AF coupling. It is found that the magnetic moment of LM remains constant over the oxidation time up to 240 sec (red line). The Ru spacer layer prevents the LM layer from further oxidation[1]. For reference, a control FM film (20 TaN | 30 Pt | 3 Co | 7 Ni | 1.5 Co | 9.5 Ru | 30 TaN) is prepared. As shown in Fig. S1b and c, the magnetic properties of the oxidized SAF (process time of 60 sec) are identical to the FM control film, thereby showing that our oxidation process affects only the upper FM layer.

Supplementary Note 2. AFM imaging and topography of FM-SAF junction

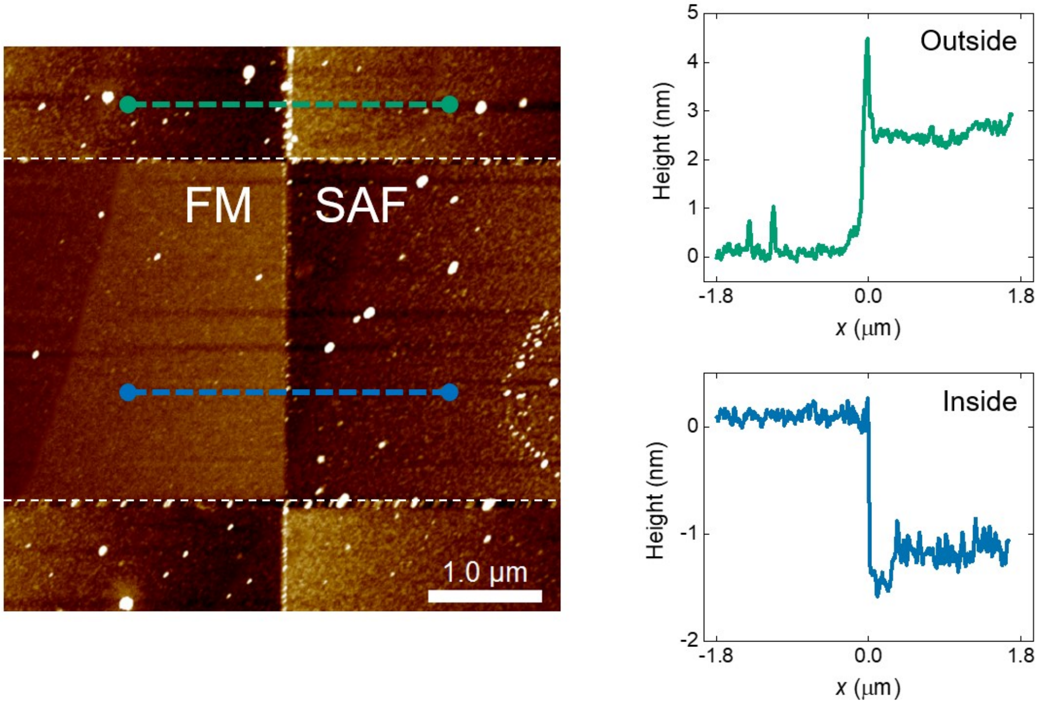

Figure S2 | Atomic force microscopy (AFM) images of FM-SAF junction ($\theta_J = 0°$) in a racetrack. The edges of the Racetrack are shown by the white dashed lines. The FM-SAF junction boundary is clearly seen from the color contrast within the nanowire. Step heights are measured on the racetrack across the junction (blue dashed line) and off the racetrack that is capsulated with alumina (green dashed line).

AFM imaging shows that the oxidation process results in a local increase in the thickness of the racetrack by ~1-2 nm as discussed in main text, which allows us to measure the width of the FM-SAF junction boundary. On the other hand, the alumina encapsulation region outside the nanowire is found to become thinner due to chemical etching of the alumina by the developer, diluted tetramethylammonium hydroxide (TMAH), as shown in Fig. S2. The etching rate of alumina is estimated to be ~ 5 nm/min at room temperature. This leads to the distinct color contrast in the optical microscopy image, as can be seen in Fig. 1a.

Supplementary Note 3. XPS depth-profile analysis of the pristine and oxidized SAF

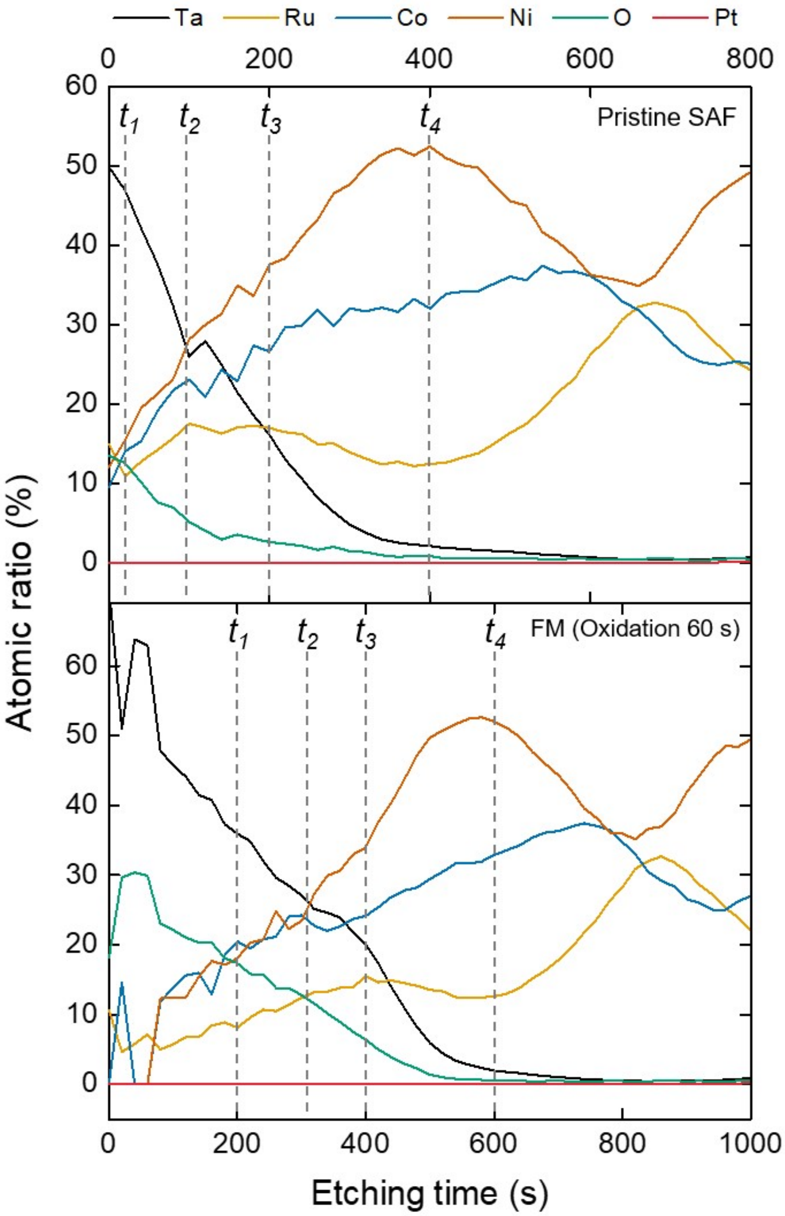

Figure S3 | XPS profile of atomic concentrations with etching time. Depth profile spectra of pristine (upper panel) and oxidized SAF (lower panel). The four times t_1, t_2, t_3, and t_4 that are shown in the figure refer to the points where the Co and Ni 2p spectra are investigated, as shown in Fig. S4.

To investigate the effect of plasma oxidation on the SAF, XPS depth profile measurements are carried out, using Ar ion etching, on the unpatterned pristine SAF film and a FM film that had been obtained by oxidization for 60 sec of a SAF film. The etching conditions are: Ar+ energy 500 eV, 0.5 μA beam current, 5 x 2 mm^2 etching area, 20 sec per cycle.

As shown in Fig. S3, it takes longer to etch the TaN capping layer for the oxidized SAF than for the pristine case, thus showing that TaN becomes thicker on oxidation by forming TaON. The depth profiles show that the oxygen level is significantly increased in the TaN, and the Co and Ni upper layers in the oxidized SAF film.

Supplementary Note 4. XPS spectra analysis of the chemical shifts in upper FM layer

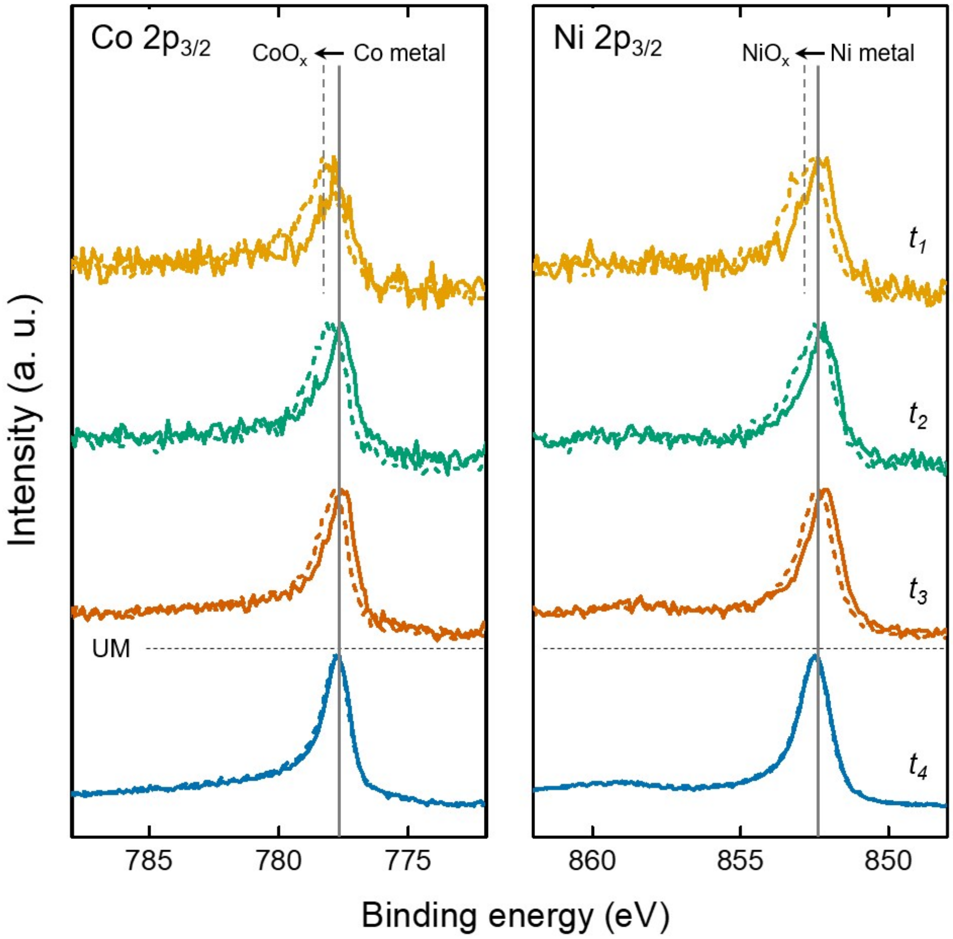

Figure S4 | XPS spectra of Co and Ni at different etching times. $2p_{3/2}$ spectra of Co (left panel) and Ni (right panel) in pristine SAF (solid line) and oxidized SAF (dashed line) are plotted at t_1, t_2, t_3 and t_4 as designated in Fig. S3. Peak positions for metallic Co and Ni are indicated, as a guide, by grey solid lines, while those from the oxidized film are displayed by grey dashed lines.

Metallic Co $2p_{3/2}$ (~778.2 eV) and Ni $2p_{3/2}$ (~852.6 eV) spectra are observed in the pristine SAF (solid line) film, thus showing that the 30 Å TaN capping layer effectively protects Co and Ni in the upper layer against natural oxidation. On the other hand, the oxidized SAF shows

chemical shifts of ~ 0.5 eV of spectral peaks towards higher binding energies, thereby confirming the oxidation of Co and Ni in the upper layer. As the film is further etched away, thus allowing analysis of the lower FM layer, Co and Ni are clearly seen to be metallic (unoxidized) (t_4).

Supplementary Note 5. Current- and field-driven DW depinning probability in FM and SAF regions

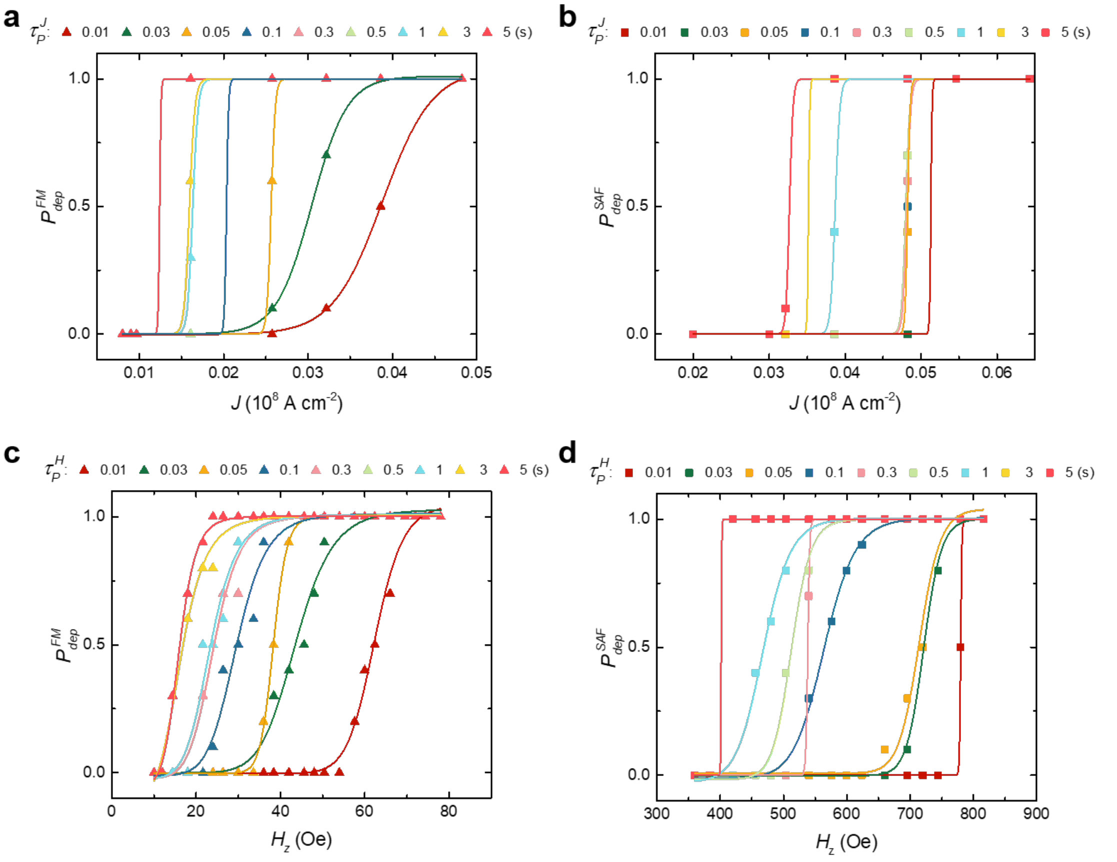

Figure S5 | Domain wall depinning probability versus current density and applied field in the thermally activated regime. a,b, Current-driven domain wall depinning probability as a function of current density in FM (a), and SAF region (b) for various current pulse lengths τ_P^J (10 to 10^4 ms). **c,d,** Field-driven domain wall depinning probability as a function of easy axis magnetic field in FM (c), and SAF region (d) for various field pulse lengths τ_P^H (10 to 10^4 ms).

To understand the domain wall (DW) dynamics in the thermally activated regime in which DW motion is dominated by thermal fluctuations, the depinning probabilities for current- and field-driven DW motion are measured inside the FM and SAF regions. Single current or magnetic easy axis field pulses are applied. The DW depinning is monitored by magneto-optical Kerr microscopy. Each depinning process is repeated 10 times. Solid lines

represent fits to the depinning probability curves. Here, the depinning threshold current density J_{th}^{i}, and field H_{th}^{i} ($i = FM$ and SAF) correspond to $P_{dep}^{i} = 0.5$ at τ_P^J, and τ_P^H, respectively[2,3].

Supplementary Note 6. Current-driven DW injection (FM → SAF) probability at FM-SAF junction

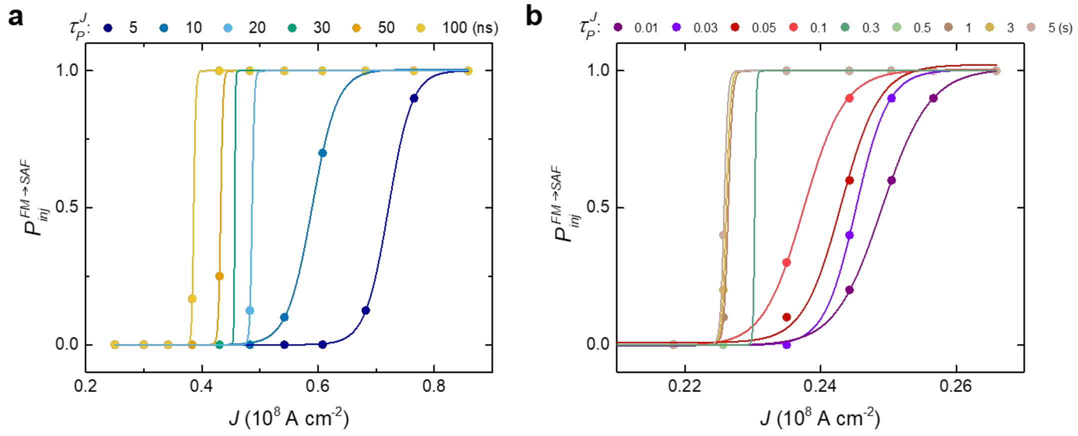

Figure S6 | DW injection (FM→SAF) probability versus current density. a,b, Current-driven DW injection probability versus current density in the flow regime (a), and the thermally activated regime (b). Current pulse lengths τ_P^J are varied from 5 to 100 ns (flow regime) and 10 to 10^4 ms (thermally activated regime) for the DW injection from FM to SAF region across the junction $\theta_J = 0°$.

Supplementary Note 7. Extraction of energy barrier in thermally activated DW motion regime

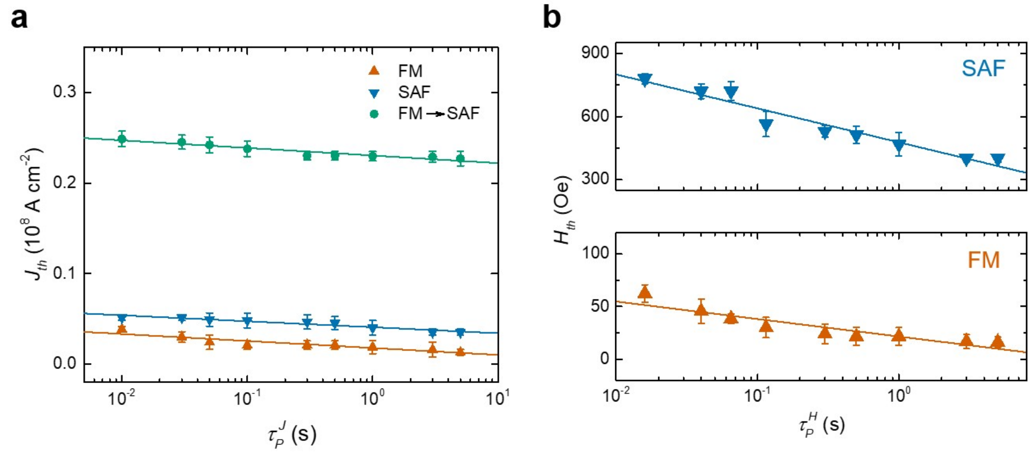

Figure S7 | Threshold current density and threshold field versus pulse length in the thermally activated regime. a, Threshold current density for DW depinning J_{dep}^{i} (i = FM and SAF) and DW injection $J_{inj}^{FM\rightarrow SAF}$ (green circle) in the thermally activated regime. J_{dep}^{i} are measured from the FM (red triangle) and SAF region (blue triangle), respectively. DW injection from the FM region into the SAF region is carried out for the junction with $\theta_J = 0°$. **b,** DW depinning threshold fields in the FM and SAF regions. H_{th}^{i} (i = FM and SAF) are measured by applying magnetic easy-axis fields for the FM (orange triangle) and SAF (blue triangle) regions. Current and field pulse lengths are varied from 10 ms to 5 s and 16 ms to 5 s, respectively. The error bars represent 25/75% probabilities.

To determine the energy barriers for DW depinning and injection, we carry out the current- and field-driven DW depinning measurements in the thermally activated regime and determine the corresponding J_{dep}^{i}, $J_{inj}^{FM\rightarrow SAF}$, and H_{dep}^{i} (i = FM, SAF and FM→SAF) as discussed in Supplementary Note 5 and 6. For DW depinning in the FM and SAF regions, DW depinning experiments were performed evenly inside each FM and SAF region, respectively, in order to exclude the effects of local pinning. According to ref. 2, 3, and 4, $\tau_P^J = \tau_0 \exp\left(E_J^{eff}/k_B T\right)$ based on the Arrhenius relation, where τ_P^J is applied current pulse length, and τ_0 is thermal attempt time. The effective energy barrier E_J^{eff} is given by $E_J^{eff} = E_J(1 -$

$J_{th}/J_{th0})^{\alpha}$ with threshold current density J_{th0} to depin DW within infinitely long time and at zero temperature. In this work, we define E_J as the energy barrier at room temperature by assuming a finite thermal attempt time $\tau_0 = 1$ ns. The exponent α corresponds to the reversal mechanism which is either $\alpha = 1$ (DW propagation dominant), or $\alpha = 2$ (DW nucleation dominant)[5,6]. Here, we employed $\alpha = 1$ since DW depinning and injection is mainly governed by propagation mechanism. By plotting an exponential fit (solid lines in Fig. S7a) with the equation $J_{th} = J_{th0}(1 - \frac{k_B T}{E_J}\ln(\frac{\tau_P^J}{\tau_0}))$ $(T = 300\ K)$, E_J can be determined. Similar to the current case, E_H can be obtained from the fits to the equation $H_{th} = H_{th0}(1 - \frac{k_B T}{E_H}\ln(\frac{\tau_P^H}{\tau_0}))$ $(T = 300\ K)$ (solid lines in Fig. S7b). H_{th} corresponds to collected threshold field H_{dep}^{i} (i = FM, and SAF) from DW depinning in each region, and H_{th0} refers to the field to depin DW within infinitely long time at zero temperature. τ_P^H is applied field pulse length, and τ_0 is thermal attempt time ($\tau_0 = 1$ ns).

Supplementary Note 8. Field-induced energy landscapes and global energy barriers for domain walls in a FM-SAF junction.

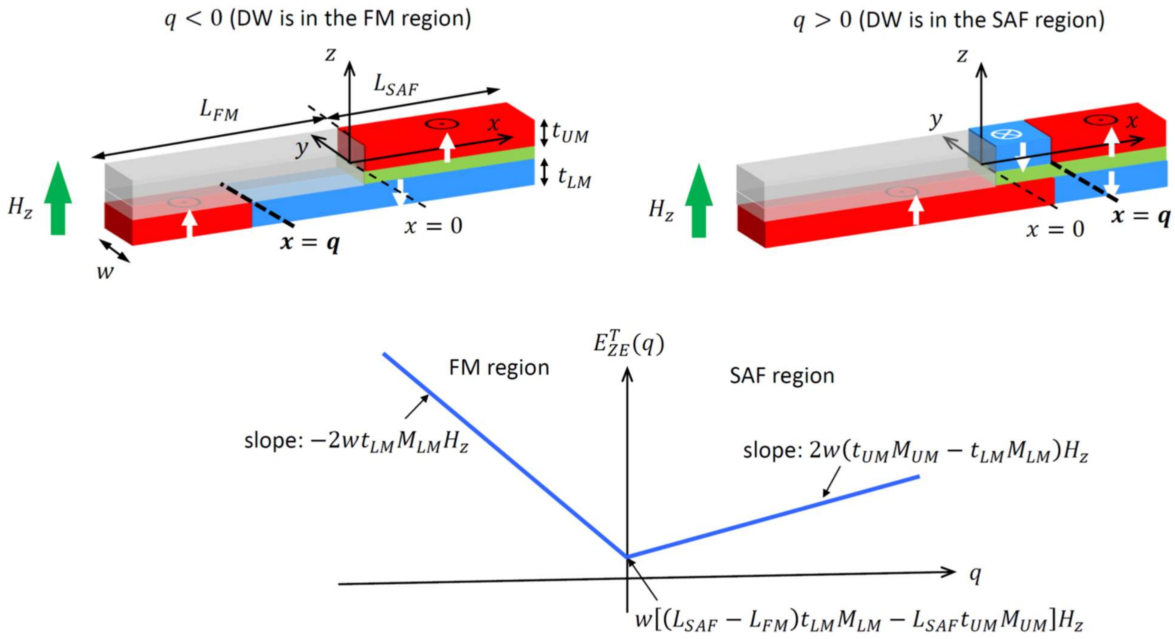

Figure S8 | Illustration of field-induced energy landscapes and global energy barriers for ↑↓ domain walls in a FM-SAF junction. Upper left panel: ↑↓ DW in the FM region ($q < 0$). Upper right panel: ↑↓ DW in the lower layer of the SAF region ($q > 0$). Lower panel: Field induced energy landscape in the FM-SAF junction with $t_{UM}M_{UM} > t_{LM}M_{LM}$.

We consider a FM-SAF lateral junction with $\theta_J = 0$ in which the junction boundary is located at $x = 0$ in a wire with length $L_{FM} + L_{SAF}$ (FM region: L_{FM}, SAF region: L_{SAF}) and width w as shown in Fig. S8. When a DW is sitting at $x = q$ in the presence of an external magnetic field $\vec{H}(x, y, z)$, the energy landscape is determined by the Zeeman energy $E_{ZE}(q) = -\int M(q, x)\hat{m}(q, x) \cdot \vec{H}(x, y, z)dxdydz$ (S1) where $M(q, x)$ and $\hat{m}(q, x)$ are the magnitude of the magnetization and the unit vector along the magnetization at x as a function of q. Note that the volume integration is valid for the magnetic layers only. The wire direction and the easy magnetization axis are along the x and z-axes, respectively. The magnetizations and thicknesses for the lower and upper magnetic layers in the SAF are M_{LM}, M_{UM}, t_{LM} and t_{UM}, respectively. Here we assume that the DW width is much smaller than both L_{FM} and L_{SAF}. When, in the presence of a uniform field H_z along the easy axis, a domain wall with ↑↓ configuration is located at $x = q$ in the FM region ($-L_{FM} < q < 0$), the energy can be written as

$$E_{ZE}^{FM}(q) = -wt_{LM}(L_{FM} + q)M_{LM}H_z - wt_{LM}qM_{LM}H_z = -wt_{LM}(L_{FM} + 2q)M_{LM}H_z. \quad \text{(S2)}$$

On the other hand, when a DW with ↑↓ in the lower layer and ↓↑ in the upper layer is sitting in the SAF region ($0 < q < L_{SAF}$), the energy in the SAF is given by

$$E_{ZE}^{SAF}(q) = -wt_{LM}qH_z + wt_{LM}(L_{SAF} - q)M_{LM}H_z + wt_{UM}qM_{UM}H_z - wt_{UM}(L_{SAF} - q)M_{UM}H_z = w(2q - L_{SAF})(t_{UM}M_{UM} - t_{LM}M_{LM})H_z. \quad \text{(S3)}$$

Hence, the total energy landscapes for the cases that the DW is located in the FM and SAF regions are, respectively,

$$E_{ZE}^{T}(q < 0: FM) = E_{ZE}^{FM}(q) + E_{ZE}^{SAF}(0) = -w[2qt_{LM}M_{LM} + (L_{FM} - L_{SAF})t_{LM}M_{LM} + L_{SAF}t_{UM}M_{UM}]H_z \quad \text{(S4a)}$$

$$E_{ZE}^{T}(q > 0: SAF) = E_{ZE}^{FM}(0) + E_{ZE}^{SAF}(q) = w[2q(t_{UM}M_{UM} - t_{LM}M_{LM}) - (L_{FM} - L_{SAF})t_{LM}M_{LM} - L_{SAF}t_{UM}M_{UM}]H_z \quad \text{(S4b)}$$

Eqs. (S4) show that, when $H_z > 0$, the slopes of energy landscapes vs. q for the cases that the DW is located in the FM ($q < 0$) and SAF regions ($q > 0$) are negative ($-2wt_{LM}M_{LM}H_z$) and positive ($2w(t_{UM}M_{UM} - t_{LM}M_{LM})H_z$), respectively, since $t_{UM}M_{UM} > t_{LM}M_{LM}$. This shows that the DW is trapped at $x = 0$ (the junction boundary) due to the formation of a global energy barrier. On the other hand, when $H_z < 0$, the signs of the slopes change so that the DW moves away from $x = 0$. For a DW with ↓↑, then the opposite is true. This notion can be extended to the SAF-FM-SAF lateral bi-junctions. The wiggles in the energy landscapes shown in Fig. 2d, 3c, 5e and Extended Data Fig. 1c illustrate the DW pinning potentials that are typically caused by non-uniform anisotropies/exchange stiffness in the films and from edge roughness of the wires[2,7]. Note that an additional large step in Fig. 2d, 3c, 5e and Extended Data Fig. 1c at the junction ($x = 0$) corresponds to the energy E_{nc} that is required to nucleate a DW in the upper layer of the SAF region.

Supplementary Note 9. Thermal depinning of domain walls in FM-SAF junctions.

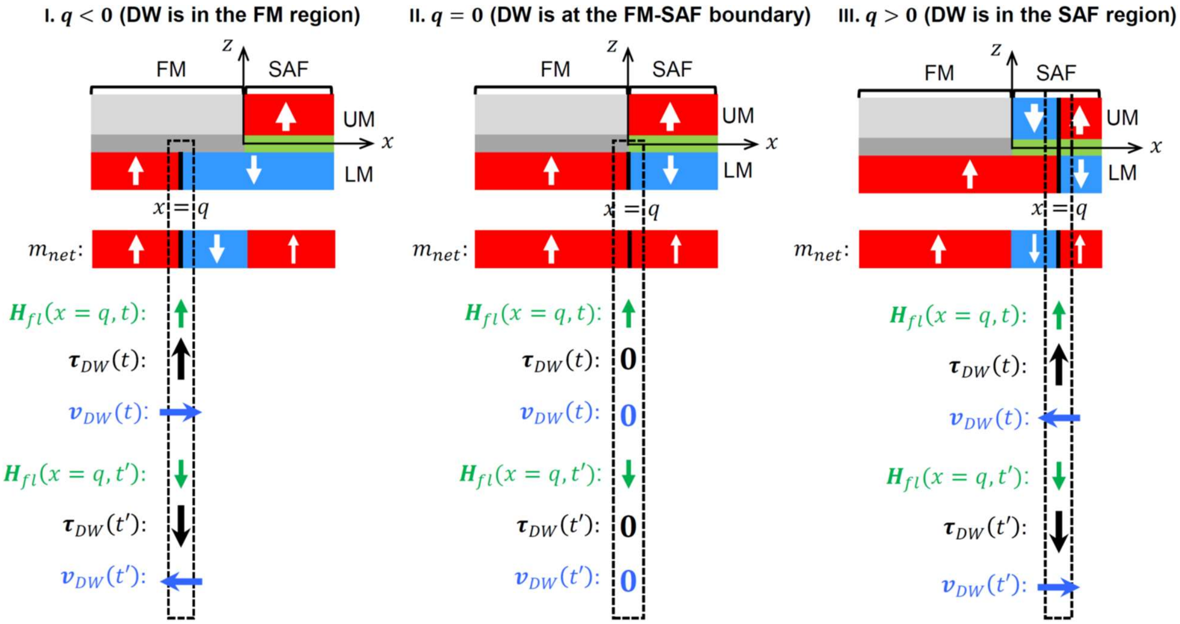

Figure S9 | Illustration of thermal fluctuation torques depending on the DW position in FM-SAF junction. **i**, ↑↓ DW in the FM region ($q < 0$), **ii**: ↑↓ DW on the boundary ($q = 0$), **ii**: ↑↓ DW in the lower layer of the SAF region ($q > 0$).

It is known that thermal depinning of domain walls is induced by thermally fluctuating magnetic fields due to microscopic degrees of freedom of the environment, such as phonons, conduction electrons or nuclear spins. Hence the thermally agitated domain wall dynamics can be described by the stochastic Landau-Lifshitz-Gilbert equation (Langevin equation)[8-10]:

$$\frac{d\boldsymbol{M}}{dt} = -\gamma \boldsymbol{M} \times \left(\boldsymbol{H_{eff}} + \boldsymbol{H_{fl}}\right) + \alpha \boldsymbol{M} \times \frac{d\boldsymbol{M}}{dt}$$

where γ, $\boldsymbol{H}_{eff}$, $\boldsymbol{H_{fl}}(\boldsymbol{r}, t)$ and α are the gyromagnetic ratio, the effective magnetic field, the random thermal fluctuating magnetic field, and the Gilbert damping, respectively. $\boldsymbol{H}_{eff}$ includes the external field, the DMI field, the exchange field, the dipolar field, and the anisotropy field. $\boldsymbol{H_{fl}}(\boldsymbol{r}, t)$ is typically assumed to be a Gaussian random process satisfying $< H_{fl}^{i}(\boldsymbol{r}, t) >= 0$ and $< H_{fl}^{i}(\boldsymbol{r}, t) H_{fl}^{j}(\boldsymbol{r}', t') >= \frac{2\alpha k_B T}{\gamma \mu_0 M} \delta_{ij} \delta(\boldsymbol{r} - \boldsymbol{r}') \delta(t - t')$ ($i, j = x, y, z$) such that $\boldsymbol{H_{fl}}(\boldsymbol{r}, t) = \boldsymbol{\zeta}(\boldsymbol{r}, t) \sqrt{\frac{2\alpha k_B T}{\gamma \mu_0 M dV dt}}$ where k_B, T, μ_0, dV, dt, and $\boldsymbol{\zeta}(\boldsymbol{r}, t)$ are the Boltzmann constant, temperature, magnetic permeability, fluctuating element volume,

fluctuating time interval, and stochastic random unit vector, respectively. δ_{ij} is the Kronecker delta. $\delta(\boldsymbol{r}-\boldsymbol{r}')$ and $\delta(t-t')$ are Dirac delta functions. dV can be approximated to be the DW volume, $wt_{FM}\pi\Delta$ (width of wire : w, FM thickness: t_{FM}, DW width parameter: Δ). If we use $\alpha = 0.1$, $T = 300$ K, $M = 600$ emu/cc, $dV = wt_{FM}\pi\Delta = 2.5 \times 10^{-17}$ cm^3 ($w = 2$ µm, $t_{FM} = 1$ nm, $\Delta = 4$ nm) and $\Delta t = 1$ ps, we have $\max\left(H_{fl}^{z}\right) \sim 177$ Oe that may depin the DW from any extrinsic potential traps in the energy landscape over an extended time in either the FM or SAF region (e.g. see Fig. 2d). Here note that only the z-component of $\boldsymbol{H}_{fl}$ has been considered since the other components (x and y) cannot depin the DWs. Note also that $\max\left(H_{fl}^{z}\right)$ cannot be directly compared to the DC DW propagation field in the FM region (~30 Oe) since the time scale for H_{fl}^{z} is ~ 1 ps.

Now let us investigate how the DWs are depinned by $\boldsymbol{H}_{fl}$ depending on where the ↑↓ DW is located (see Fig. S9). When $q < 0$ (case I. DW is in the FM region) or $q > 0$ (case III. DW is in the SAF region), the DW is depinned by $\boldsymbol{H}_{fl}$ that is applied to the DW at $x = q$ and at a certain time t. The directions of the depinning torque $\boldsymbol{\tau}_{DW}$ and the resulting DW velocity $\boldsymbol{v}_{DW}$ are determined by the sign of $H_{fl}^{z}(q,t)$ as shown in Fig. S9. For example, when $q < 0$ (FM region) and $H_{fl}^{z}(q,t) > 0$, $\tau_{DW}^{z} > 0$ and $v_{DW}^{x} > 0$ (case I in Fig. S9). When $q > 0$ (SAF region) and $H_{fl}^{z}(q,t) > 0$, $\tau_{DW}^{z} > 0$ and $v_{DW}^{x} < 0$ since in the SAF region the upper layer moment is larger than the lower layer moment such that the DW configuration is ↓↑ as seen from the m_{net} landscape (case III in Fig. S9). As $H_{fl}^{z}(q,t)$ changes sign, then these statements are vice versa for both the FM and the SAF regions.

In contrast, when $q = 0$ (case II. DW is at the FM-SAF boundary), the situation is distinct from the others: there is effectively no DW (see the net magnetic moment m_{net} landscape in case II in Fig. S9). This is a very special case, thus corresponding to a singular point at which the whole wire is nothing but a single domain due to the fact that the upper moment is larger than the lower moment in the SAF region. This suggests that any direction of $\boldsymbol{H}_{fl}$ cannot depin or move the DW at $q = 0$ unless a new DW is created, thus showing that the DW at the FM-SAF boundary is extremely thermally stable.

Supplementary Note 10. Domain wall propagation from FM to SAF region in the tilted FM-SAF junctions

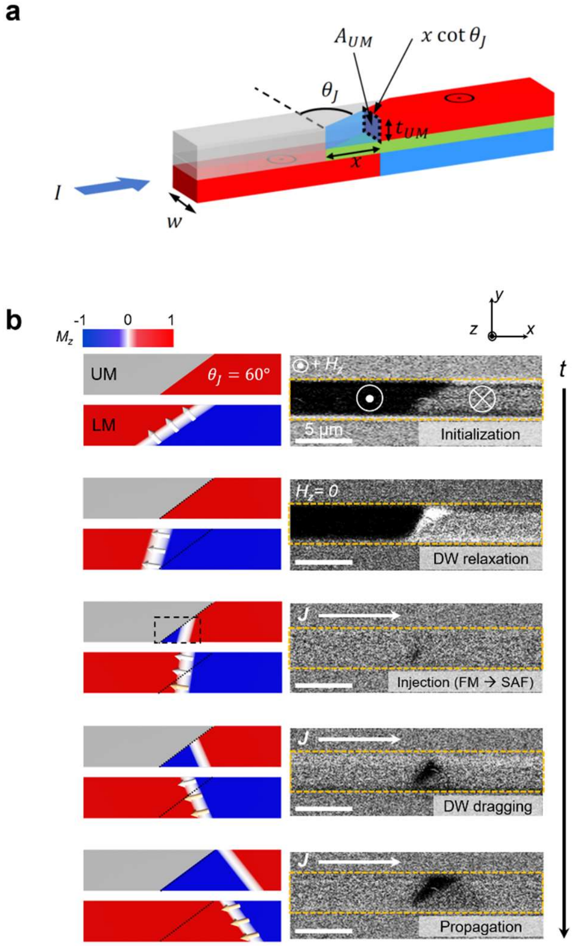

Figure S10 | Domain wall injection into tilted FM-SAF junctions. a, Illustration of domain wall propagation from FM to SAF region in the tilted FM-SAF junctions. **b,** Schematic illustrations and Kerr microscope images of domain wall injection from FM to SAF region at $\theta_J = 60°$.

Let us assume that the DW cross sectional area A_{UM} in the upper layer is $A_{UM} = t_{UM} x \cot \theta_J$ where x is the distance between the junction endpoint and the DW (see Fig. S10a). As the DW propagates (i.e. x increases) reaching $x = w \tan \theta_J$ in the tilted junction region, $A_{UM} = t_{UM} w$. Consequently, we find that $\frac{dA_{UM}}{dx} = t_{UM} \cot \theta_J$ thereby showing that the larger θ_J is the smaller is $\frac{dA_{UM}}{dx}$. This means that the gradient of DW volume to be nucleated decreases with increasing θ_J, as the DW propagates in the junction region, consequently making it easier

for the DW to propagate at larger θ_J. This is clearly shown in experiments as shown in Fig. S10b. At $\theta_J = 60°$, the DW can be initialized by following the shape of junction in the presence of external field (DW initialization). However, the DW relaxation is observed and the shape of DW becomes straight which is perpendicular to the wire as soon as the field is vanished (DW relaxation). Afterwards, the DW can be injected from the position where the DW faces to the junction area (black dashed box) and propagates into SAF region (injection).

Supplementary Note 11. Sequential multi-DW initialization and injection in FM-SAF junction

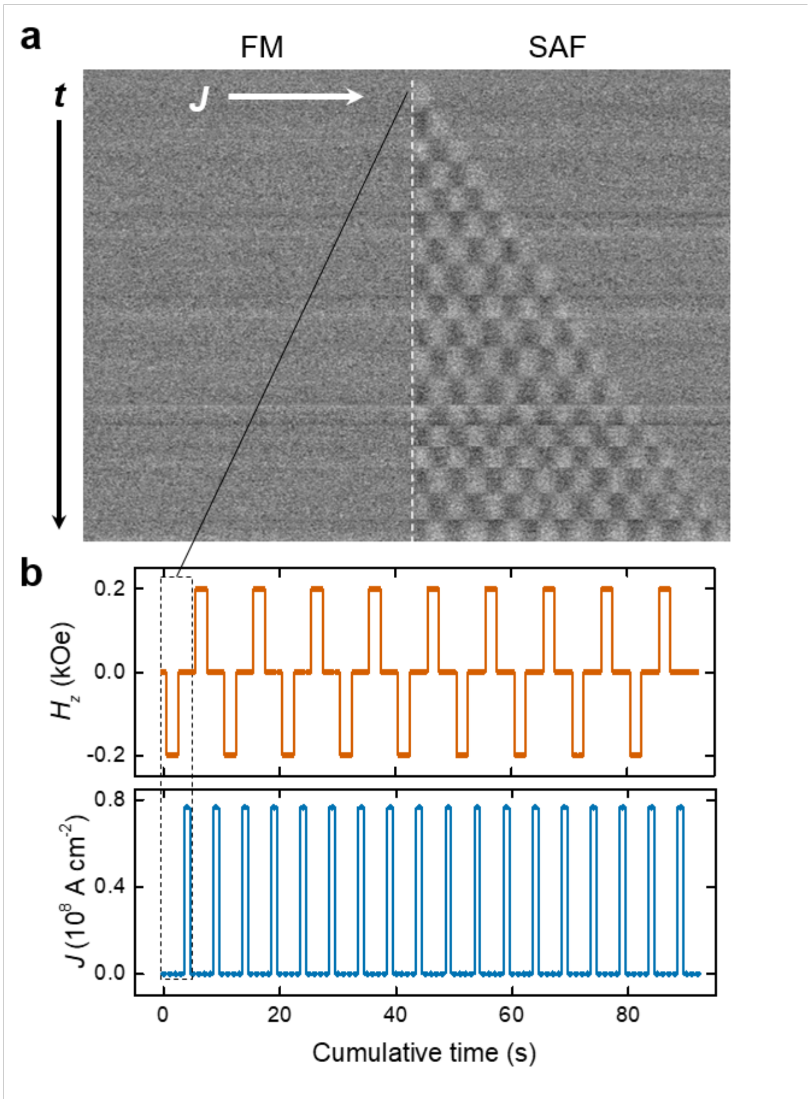

Figure S11 | Sequential DW initialization and multi-bit injection from the FM into the SAF region in a racetrack. a, Sequential Kerr microscopy images of injection of eighteen DWs from the FM region into the SAF region. The racetrack is 3 µm wide and 40 µm long. The FM-SAF junction with $\theta_J = 0°$ is designated by the white dashed line. Each Kerr image is taken after a combination of field and current pulse applications. Bright and dark contrast correspond to down (↓) and up (↑) domains, respectively. **b,** Cycles of field and current pulses versus cumulative time corresponding to Kerr images in (a). Each cycle is denoted by the black dashed line, which is repeated eighteen times.

By taking advantage of the large difference in coercivity and spin-flop field between the FM and SAF regions, DWs can be readily and reliably created by applying an external field. The field $|H_z|$ is chosen to be $|H_c^{FM}|(\approx 0.1\ \mathrm{kOe}) < |H_z| < |H_{sf}^{SAF}|(\approx 3\ \mathrm{kOe})$ such that a DW is nucleated in the FM region. The created DW is injected into the SAF region by current pulses. By repeating the DW nucleation and injection cycle, we demonstrated multi-DW injection, as shown in Fig. S11a. Each cycle for DW injection consists of a field pulse of $\pm$ 20 mT for 2 sec and a current pulse of $0.76\ \times\ 10^8$ A cm^{-2} for 10 ns (see Fig. S11b). An external field is set to zero when the current pulse is applied. Consequently, eighteen DWs can be successfully injected with wall-to-wall distances of ~1.4 μm. The domain bit sizes can be modulated by the amplitude and length of the current pulse.

Supplementary Note 12. Determination of DW injection across the FM-SAF junction

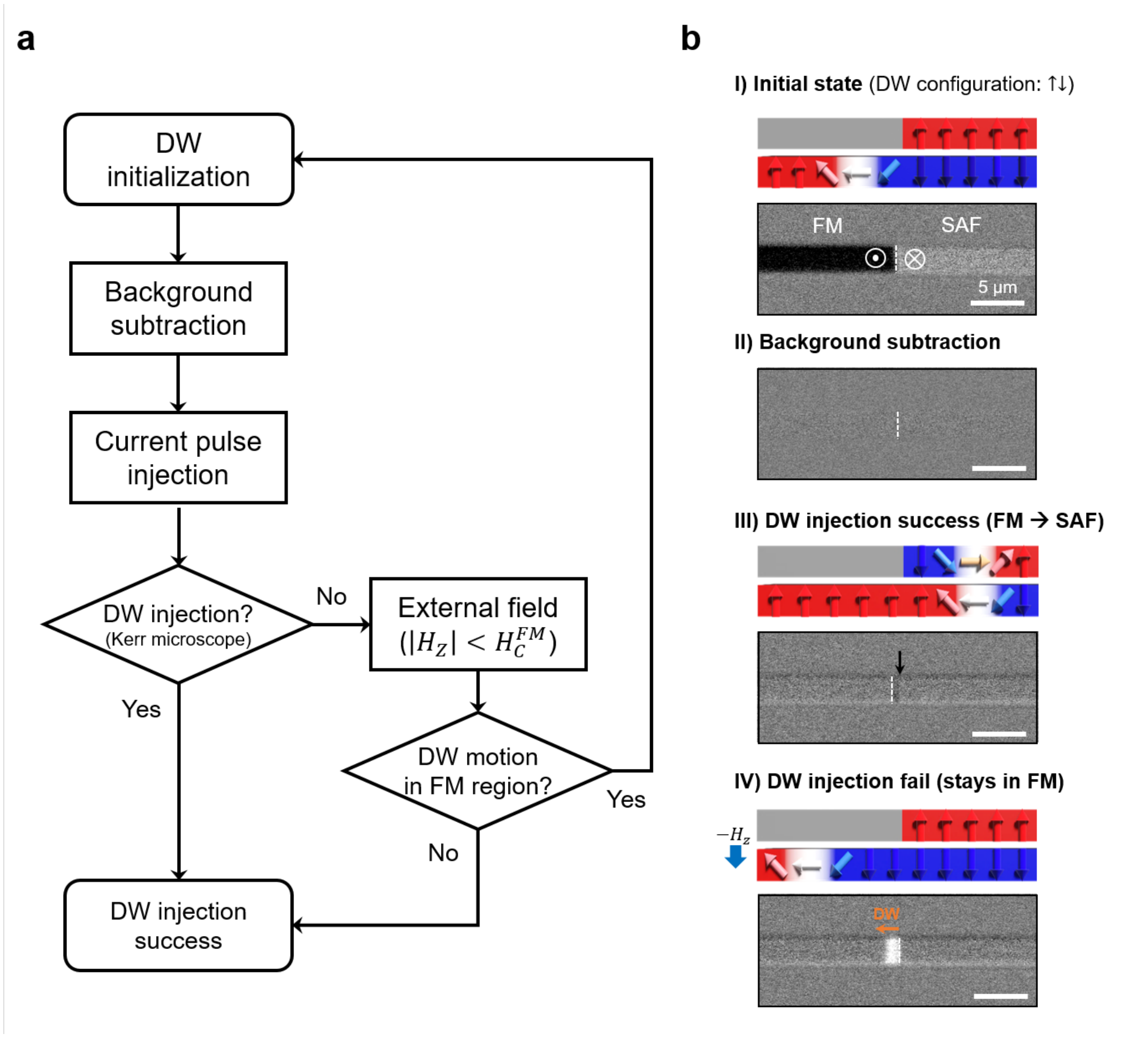

Figure S12 | Determination procedure of DW injection across a FM-SAF junction. a, Logic flow diagram of determination procedure of DW injection from FM to SAF region across junction by current pulse. **b,** Schematics and corresponding Kerr microscope images of DW injection procedure. White dashed lines represent the FM-SAF junction, and black arrows indicate the DW injected into the SAF region.

The optical resolution of a Kerr microscope limits the confirmation of DW injection across the interface. Fortunately, our FM-SAF junctions with their novel magnetic configuration by design allows us to carry out extremely precise and reliable confirmation of DW injection across the interfaces by combining Kerr microscopy with the application

of easy axis magnetic fields that are larger than the DW propagation fields, as follows. Here let us take a ↑↓ DW at the FM-SAF junction in which the FM and the SAF regions are on the left and right-hand side, respectively.

a. When the DW is observed to be clearly injected across the interface from Kerr microscope, we are sure that the DW is injected since the DW has been displaced by more than the Kerr microscope resolution.
b. When the DW is observed not to be injected by current pulse from Kerr microscope, an easy axis magnetic field is applied to check if the DW is actually injected across the interface. First, let us consider a DW that is initially in the FM region at the FM-SAF junction. If the DW was successfully injected across the interface so that it moves to the SAF region, the application of a negative H_z ($-H_z < 0.4$ kOe: propagation field in the SAF region) would move the DW from the left to the right since the total DW configuration in the SAF region is ↓↑ (Note that the upper layer moment is larger than the lower layer). On the other hand, if the DW fails to be injected so that it still sits in the FM region, a negative H_z ($-H_z < 30$ Oe: propagation field in the FM region) would move the DW from the right to the left. Consequently, the DW moving direction by a negative H_z is opposite depending on whether the DW is successfully injected across the interface or not, from which we can rigorously judge the DW injection. Since the propagation field in the SAF region (~0.4 kOe) is significantly larger than the FM region (~30 Oe), the application of H_z in-between two propagation fields, i.e., $30\text{ Oe} < -H_z < 0.4$ kOe can also tell whether the DW is injected across the interface. Fig. S12b describes this method to judge the DW injection across the interface.
c. The same protocol as above can be applied to the case that the DW is injected from the SAF into the FM region.

Supplementary Note 13. The energy barriers for field and current-driven DW motion

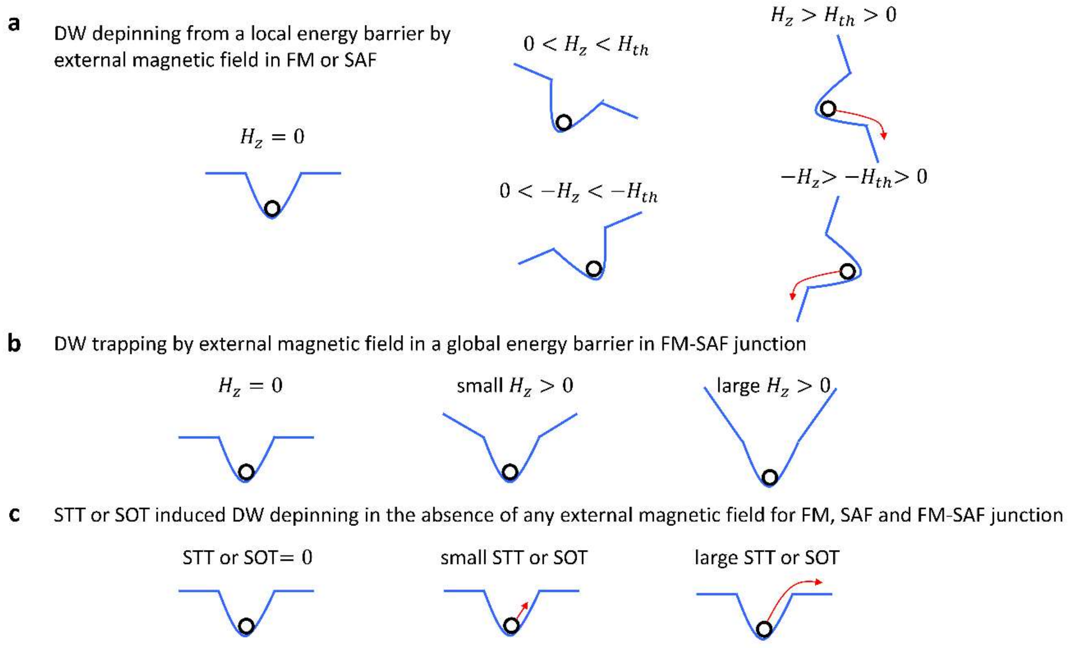

Figure S13 | Illustration of DW depinning and energy landscapes for ↑↓ DW configuration. a, DW depinning from a local energy barrier by external magnetic field for the FM and SAF cases. **b**, DW trapping by external magnetic field in a global energy barrier for a FM-SAF junction. **c**, STT or SOT induced DW depinning in the absence of external magnetic fields including thermal fluctuation induced magnetic fields for FM, SAF and FM-SAF junction.

Let us clarify the field-, the STT- and SOT-driven DW motions. The STT and SOT are generated by current. The DW angle corresponds to the azimuthal angle ϕ since the polar angle $\theta = \frac{\pi}{2}$ in the middle of DW. Importantly, note that the energy landscape and barrier is a function of DW position q solely by definition as shown in Fig. S13 above.

a. Field-driven DW motion

The reviewer is right that in the steady state the DW angle ϕ does not change but the DW position defined by θ changes by an easy axis component of field. For the FM or SAF case, an easy axis field that is larger than a threshold value H_{th} can displace DWs by tilting the DW energy landscape and thereby overcoming the local energy barrier (see Fig. S13a below, and note that field components perpendicular to the easy axis do not displace the DWs since they do not affect the energy landscapes). The key point here is that the global energy landscape is

uniformly tilted over the whole FM or SAF, which just allows the DW to get out of the local energy barrier. This means that the global energy landscape does not give rise to an additional energy barrier in addition to the local barrier.

In sharp contrast, for the FM-SAF junction the easy axis field fundamentally modifies the global energy landscape, thus adding a global energy barrier to the local barrier, as shown in Fig. S13b. This strongly and robustly traps the DW near the junction boundary.

b. STT-driven DW motion

STT drives a DW by the law of angular momentum conservation without affecting the energy landscape as shown in Fig. S13c. Thus, the torque induced by the transfer of spin angular momentum from the moving electrons into the DW moments actively changes the polar angle θ, thereby directly displacing and driving the DW[11].

c. SOT-driven DW motion

SOT drives a DW by a combined mechanism of Dzyaloshinskii-Moriya interaction (DMI) induced in-plane field and spin-orbit spin-current induced STT[11]. Here the spin-orbit spin-current induced STT changes the DW angle ϕ. This increases the magnetic energy against the DMI, thereby changing θ and displacing the DW. Hence, the SOT does not affect the energy landscape or barrier just like the STT case, as shown in Fig. S13c.

Supplementary Note 14. Evaluation of pulsed magnetic field

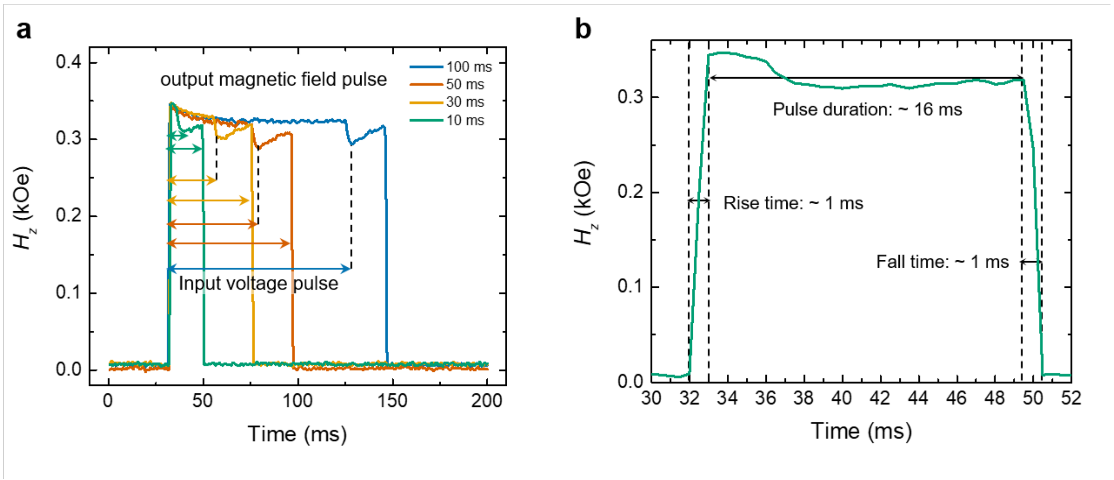

Fig. S14. Profiles of pulsed magnetic fields measured by Hall probe. a, Measured magnetic field pulses with a magnitude of $H_z \sim 0.35$ kOe and corresponding input voltage pulse duration from 10 to 100 ms. Black dashed lines and arrows correspond to the applied pulse duration, respectively. **b,** Shape of single pulse magnetic field with 10 ms duration.

Thermally assisted field-driven DW motion in FM and SAF regions were performed by applying single pulsed magnetic fields along the magnetic easy axis with a broad range of pulse durations τ_P^H from 10 ms to 5 s. Single pulses of magnetic field are generated by an Evico electromagnetic inductor coil combined with a Kepco bipolar BOP 100-4D power supply and a Keithley 2400 pulse generator.

To evaluate the shape of the magnetic field pulse, a Hall probe sensor combined with a digital oscilloscope were employed to measure the pulse shape resulting from the Hall voltage in response to the magnetic field changes. As shown in Fig. S14a, the shape of the field pulses with different pulse lengths exhibit a rise and fall time, each of ~ 1 ms, and a pulse duration corresponding to the nominal input voltage pulse length of from 10 to 100 ms (see arrows). Contrary to the nominal input pulse length, the actual magnetic field pulse length is slightly longer. For instance, the 10 ms nominal pulse length has a measured length of ~16 ms, as shown in Fig. S14b.

Supplementary Note 15. Effect of electrical current fluctuations on DW depinning

At finite temperatures, electrical current fluctuations induced by thermal effects may depin DWs if the fluctuations are large enough to overcome the local energy barrier even in equilibrium. Here we quantify the magnitudes of such fluctuations to investigate how they affect the DW motion and depinning by following ref. 3 based on the fluctuation-dissipation theorem.

We assume here that the current density randomly fluctuates with a magnitude of δJ with respect to the applied current density J, and independently of each other over a time scale of τ. In typical metallic systems, the Drude model shows that a temporal fluctuation of J decays within $\tau \sim 10^{-15} - 10^{-13}$ sec that corresponds to the electron scattering time. The averaged magnitude δJ is derived from the spectral density function that is given by

$$S_J(\omega) = \frac{4k_BT}{V} \cdot Re\sigma(\omega) \quad (\omega > 0) \tag{S5}$$

where $\sigma(\omega)$ is the electrical conductivity at frequency ω, k_B is the Boltzmann constant, T is the temperature, and V is the volume of sample. If a DW is depinned within the time Δt that is much larger than τ, we obtain the average magnitude δJ by integrating Eq. S1 with respect to ω, for the interval $0 < \omega < \pi/\Delta t$ as follows:

$$\delta J = \sqrt{\frac{k_BT}{V} \cdot \frac{\sigma}{\Delta t}} \qquad (0 < \omega < \pi/\Delta t) \tag{S6}$$

where σ is the d.c. conductivity ($\omega = 0$). Using $T = 300$ K, $\sigma \sim 1.28 \times 10^6 \Omega^{-1}\mathrm{m}^{-1}$, DW volume $V_{DW} \sim 2.5 \times 10^4$ nm^3 and $\Delta t = 1$ ns, we obtain $\delta J \cong 4.5 \times 10^4$ A/cm^2. These values are much smaller than $J_{th}^i \sim 10^7$ A/cm^2 by more than two orders of magnitude (*i* = FM, SAF, and FM → SAF), thus showing that the DW depinning induced by thermally driven current density fluctuations is negligible.

Following ref. 3, we estimate the DW depinning probability *P* induced by current density fluctuations within a long period of time *t* as follows:

$$\ln P \sim -\frac{(J/\delta J)^2}{2} + \ln(t/\Delta t). \tag{S7}$$

If we take $J_{th}^{SAF} \sim 3.0 \times 10^7$ A/cm^2, $\delta J \cong 4.5 \times 10^4$ A/cm^2, $t = 10^{10}$ sec, and $\Delta t = 1$ ns, we obtain $P \sim e^{-2.2\times 10^5} \sim 10^{-10^5}$. Consequently, the depinning probability by δJ is eventually zero over a 300 year ($\cong 10^{10}$ sec) time period.

Supplementary References

1. Balcerzak, J., *et al.*, In-situ XPS analysis of oxidized and reduced plasma deposited ruthenium-based thin catalytic films. *Applied Surface Science* **426**, 852-855 (2017).
2. Kim, K.-J. *et al.* Two-barrier stability that allows low-power operation in current-induced domain-wall motion. *Nature Communications* **4**, 2011 (2013).
3. Fukami, S., Yamanouchi, M., Ikeda, S. & Ohno, H. Depinning probability of a magnetic domain wall in nanowires by spin-polarized currents. *Nat Commun* **4**, 2293 (2013).
4. Khvalkovskiy, A.V., *et al.,* Basic principles of STT-MRAM cell operation in memory arrays. *J. Phys. D: Appl. Phys.* **46**, 074001 (2013).
5. Koch, R.H., Katine, J.A., and Sun, J.Z., Time-resolved reversal of spin-transfer switching in a nanomagnet. *Phys. Rev. Lett.* **92**, 088302 (2004).
6. Raquet, B. et al., Dynamical properties of magnetization reversal in an ultrathin Au/Co film. *J. Magn. Magn. Mater.* **150**, L5-L12 (1995).
7. Garg, C., Yang, S.-H., Phung, T., Pushp, A. & Parkin, S. S. P., Dramatic influence of curvature of nanowire on chiral domain wall velocity. *Science Advances* **3**, e1602804 (2017).
8. Martinez, E., Lopez-Diaz, L., Torres, L., Tristan, C. & Alejos, O. Thermal effects in domain wall motion: Micromagnetic simulations and analytical model. *Phys. Rev. B* **75**, 174409 (2007).
9. Moretti, S., Raposo, V. & Martinez, E. Influence of Joule heating on current-induced domain wall depinning. *Journal of Applied Physics* **119**, 213902 (2016).
10. Brown, W. F. Thermal Fluctuations of a Single-Domain Particle. *Phys. Rev.* **130**, 1677–1686 (1963).
11. Yang, S.-H., and Parkin, S. S. P., Novel domain wall dynamics in synthetic antiferromagnets. *J. Phys.: Condens. Matter* **29**, 303001 (2017)
